# Grand Challenges and Opportunities in Stimulated Dynamic and Resonant Catalysis

**DOI:** 10.1021/acscatal.5c07014

**Published:** 2026-02-11

**Authors:** Matteo Monai, Wiebke Albrecht, Achim Alkemper, Nongnuch Artrith, Andrea Baldi, Arik Beck, Ryan T. Berry, Ettore Bianco, Floor A. Brzesowsky, Qi Dong, Jimmy A. Faria Albanese, Renee R. Frontiera, Elaina Galvin, Erik C. Garnett, Nick Gerrits, Marek Grzelczak, Marc Herzog, Franziska Hess, Alexander A. Kolganov, Wouter Koopman, Nikolay Kosinov, Sarah Lander, Enrico Lepre, D. Nicolette Maaskant, Guobin Miao, Aadesh Mohan Naik, Tzia Ming Onn, Andrew A. Peterson, Diana Piankova, Evgeny A. Pidko, Korawich Trangwachirachai, Floris van den Bosch, Di Xu, Begum Yilmaz, Johannes Zeininger, Esther Alarcón Lladó, Jörg Meyer, Paul J. Dauenhauer, Sven H. C. Askes

**Affiliations:** † Inorganic Chemistry and Catalysis Group, Institute for Sustainable and Circular Chemistry, 84889 Utrecht University, Utrecht 3584 CG, The Netherlands; ‡ 55952 AMOLF, Amsterdam 1098 XG, The Netherlands; § Institute for Material Science, Technical University Darmstadt, Darmstadt 64287, Germany; ∥ Debye Institute for Nanomaterials Science, 393819 Utrecht University, Utrecht 3584 CC, The Netherlands; ⊥ Department of Physics and Astronomy, 1190 Vrije Universiteit Amsterdam, Amsterdam 1081 HV, The Netherlands; # The Institute for Chemical Technology and Polymer Chemistry, 150232 Karlsruhe Institute of Technology, Karlsruhe 76131, Germany; ∇ Center for Programmable Energy Catalysis, Dow Discovery Fellowship, Department of Chemical Engineering, University of California Santa Barbara, Santa Barbara, California 93106, United States; ○ Department of Chemistry and NIS Centre, 9314 University of Torino, Torino 10125, Italy; ◆ Department of Chemistry, 1068 Purdue University, West Lafayette, Indiana 47907, United States; ¶ Department of Chemical Engineering, 3230 University of Twente, Enschede 7522 NB, The Netherlands; ⟁ Department of Chemistry, 5635 University of Minnesota, Minneapolis, Minnesota 55455, United States; △ Leiden Institute of Chemistry, 4496 Leiden University, Leiden 2333 CC, The Netherlands; ▲ 16379 Centro de Fisica de Materiales (CFM-MPC), CSIC-UPV/EHU, Paseo Manuel de Lardizabal 5, Donostia 20018, Spain; ▽ Institute of Physics and Astronomy, 26583 University of Potsdam, Potsdam 14476, Germany; ▼ Institute of Chemistry, 26524 Technical University Berlin, Berlin 10623, Germany; ◬ Department of Chemical Engineering, 2860 Technical University of Delft, Delft 2629 HZ, The Netherlands; ◮ Department of Chemical Engineering and Chemistry, 3169 Eindhoven University of Technology, Eindhoven 5612 AZ, The Netherlands; ◭ Department of Chemistry, University of Zürich, Zürich 8057, Switzerland; ⧩ Department of Engineering, 2152 University of Cambridge, Cambridge CB2 1PZ, U.K.; ⧨ School of Engineering, 6752 Brown University, Providence, Rhode Island 02912, United States; ◁ Department of Energy Conversion and Storage, Technical University of Denmark, Kongens Lyngby 2800, Denmark; ◀ Department of Mechanical and Process Engineering, 27219 ETH Zürich, Zürich 8093, Switzerland; ▷ Inorganic Systems Engineering, Department of Chemical Engineering, Faculty of Applied Sciences, Delft University of Technology, Delft 2629 HZ, The Netherlands; ▶ Department of Chemical & Biomolecular Engineering, 37580 National University of Singapore, Singapore 117585, Singapore; ⧅ Institute of Materials Chemistry, 27259 Technical University Vienna, Vienna 1060, Austria; ⧄ NWO-Institute AMOLF, Amsterdam 1098 XG, The Netherlands; ⊞ van’t Hoff Institute for Molecular Sciences, University of Amsterdam, Amsterdam 1090 GD, The Netherlands; ◘ Center for Programmable Energy Catalysis, Department of Chemical Engineering and Materials Science, 5635 University of Minnesota, Minneapolis, Minnesota 55455, United States

**Keywords:** stimulated catalysis, dynamic catalysis, resonant catalysis, stimulando characterization, light, strain, temperature swing, electric charge

## Abstract

Traditional heterogeneous catalysis is constrained by kinetic and thermodynamic limits, such as the Sabatier principle and reaction equilibrium. Dynamic and resonant catalysts hold promise to overcome these limitations by actively oscillating a catalyst’s physical or electronic structure at the time scale of the catalytic cycle, allowing programmable control over reaction pathways, and leading to improved rate and selectivity. External stimuli such as temperature swing, mechanical strain, electric charge, and light can perturb catalyst surfaces in different ways, altering adsorbate coverage, binding energies, and transition states beyond what steady-state catalysis allows. This work surveys the current state of dynamic catalysis, introduces the concept of *“stimulando”* characterization for observing transient dynamics, and outlines key modeling, mechanistic, and benchmarking strategies to advance the field toward improved chemical transformation.

## Graphical abstract

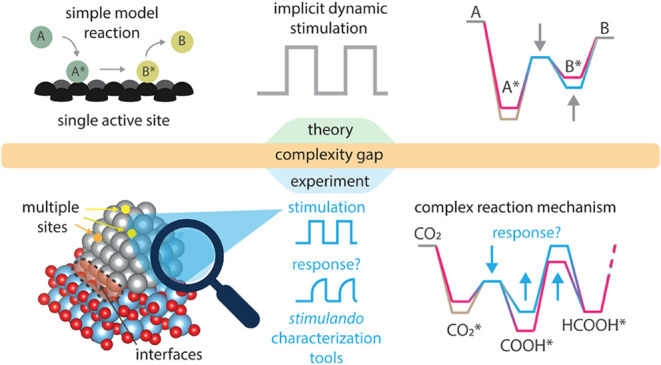

## 1 Introduction

Opportunity for improved control of chemistry exists with a new class of dynamic and resonant catalysts that change on the time scale of surface chemistry. Conventional heterogeneous catalysis of the past century has focused on designing catalyst surfaces, assuming that active sites are static over a complete catalytic turnover.^1^ This catalyst engineering strategy focusing on structure–function relationships has primarily advanced via increasing the fundamental description of active site mechanisms. Deeper knowledge of catalytic mechanisms provides insight into the rational design of faster, more selective catalyst structures and compositions.^2,3^ However, this “deep knowledge” catalysis design philosophy is limited by the inherent capabilities of static active sites, which have fundamental restrictions such as the Sabatier limit or reaction equilibrium; static catalysts can only accelerate reactions up to a maximum peak rate and to a conversion defined by equilibrium.^4,5^

The ability to oscillate the electronic or physical structure of a catalyst’s active site introduces new capabilities for controlling surface reactions.^6^ As depicted in Figure 1a, dynamic surfaces result in multiple free energy pathways between reactant, A­(g), and product, B­(g), through intermediates A* and B* and their connecting transition state, TS.^7^ The catalyst is forced to change via energy input referred to here as “stimulus”, which can be imposed on the surface by a variety of mechanisms. The magnitude of catalyst stimulation can be described in terms of the perturbation mechanism (e.g., applied light frequency) or as the extent of change in binding energy of an adsorbate, ΔBE_i_ for species i, resulting from the stimulus.

**Figure 1:**
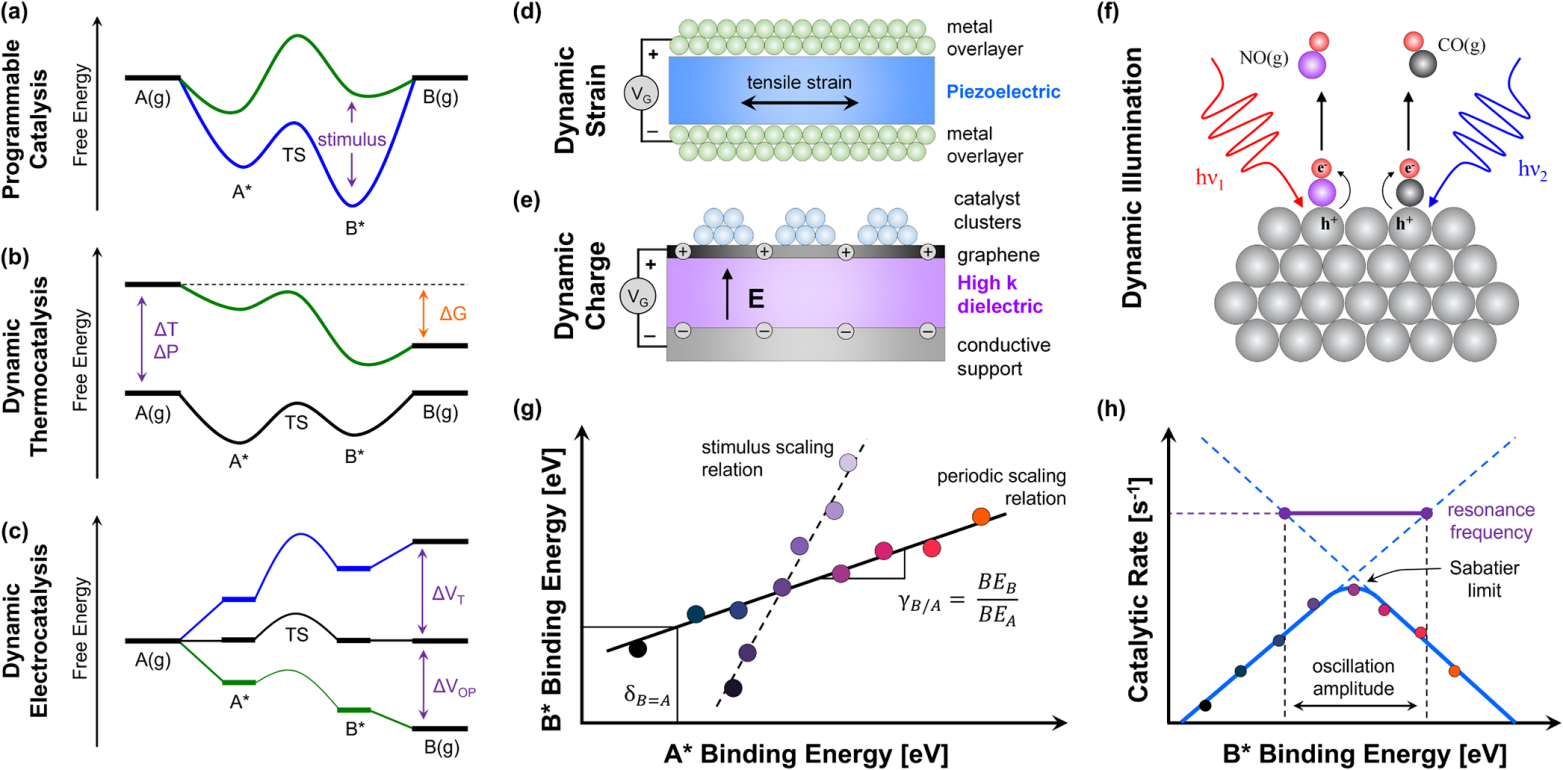
Stimulated and resonant catalysis: concept and scope. (a) Programmable catalysts are surfaces and active sites that change in physical and/or electronic state via a controlled external stimulus with defined temporal perturbations in the binding energy of adsorbates, A* and B*. (b,c) Oscillation of temperature (*T*), pressure (*P*), composition (*X*), or overpotential (*V*_OP_) are alternative methods of changing the overall thermodynamics and resulting kinetics of reactions. Methods of stimulating catalysts to shift active site electron density and the binding energy of adsorbates include: (d) tensile and compressive strain and stress via devices such as support piezoelectric materials, (e) charge condensation in catalyst active sites via the condenser device architecture, or (f) pulsed illumination of catalyst surfaces. (g) Different catalytic materials (e.g., Pt, Ru, Rh) exhibit periodic linear scaling between the binding energy of adsorbates (A* and B*), while external stimuli shift the binding energy of molecules on each individual material. (h) Linear scaling of adsorbate binding results in a Sabatier “volcano” (blue) with a peak maximum rate. Oscillation of the binding energy of B* for any given amplitude accelerates the reaction at a “resonance frequency” above the Sabatier maximum.

When stimuli can be applied at controlled frequencies (*f*), the catalyst is referred to as “programmable”; the catalyst active sites are forced to change as defined by the input program, which is engineered in extent and speed of active site changes to optimize for reaction rate, selectivity, and/or conversion. Furthermore, because the stimulation can provide surface work by changing the BE of intermediates in so-called “catalytic ratchets”,^7^ reactions can be promoted over traditional static thermodynamic boundaries, similarly to the ratcheting mechanisms that drive reactions in biological systems. For example, microkinetic modeling of dynamically stimulated Ru catalysts predicted an enhancement in N_2_ conversion to NH_3_ via the Haber-Bosch reaction beyond the static equilibrium limit.^8^ This showcases the potential of programmable and stimulated catalysis for chemical transformation.

Catalyst dynamics that vary the binding energy of adsorbates have recently become possible due to new methods of applying stimuli directly to catalyst surfaces, making them distinctly different from changes in reactor temperature, pressure, and composition (Δ*T*, Δ*P* in Figure 1b) or overpotential (Δ*V*_OP_ in Figure 1c) that have been studied for decades.^6^ Methods of stimulating catalyst surfaces are primarily variations of modulating the physical and/or chemical properties of an active site (e.g., strain, temperature, electron density).^6^ Physical deformation of surfaces can occur by methods that apply pressure waves to the surface or utilize catalyst supports that deform with time via external control, such as the case of a piezoelectric support (Figure 1d) that expands and contracts with an applied voltage.^9,10^ Alternatively, electrons or holes can be accumulated at an active site by methods such as a catalytic condenser (Figure 1e) that achieves high charge density (>10^13^ electrons/cm^2^) using high-capacitance oxide films.^11,12^ Third, photons incident on the catalyst surface (Figure 1f) can provide both thermal and nonthermal energy that has been shown to generate electron–hole pairs and increase the rate of reaction or desorption.^13^ These stimuli affect specific adsorbate–surface interactions such that each method of perturbing catalyst surfaces will provide distinct changes in adsorbate binding energies and transition states.

The stimulation of reactive surfaces exhibits predictable molecular behavior that helps in the design of dynamic catalysts. The variation in the binding of adsorbates on different catalyst materials and active sites has historically been described via linear scaling relationships that account for the differences between any two surface species.^14^ As an example shown in Figure 1g, the generic adsorbates A* and B* both increase in binding energy to different extents, described by the slope, γ, as a trend on many materials. Stimulation of a catalyst surface with light, charge, or strain also varies the binding energy of molecules on the surface of a single material, albeit at a unique slope from periodic scaling; the ability of a stimulus to shift the binding energy proportional to the extent of the applied perturbation is the key capability of dynamic and programmable catalysis. Similar linear scaling relationships, such as the Brønsted–Evans–Polanyi (BEP) relation, describe the energy of the transition state between two adsorbates.^15^ These descriptors for molecules on catalyst surfaces lead to the formation of a Sabatier volcano, depicted in Figure 1h, where the catalytic rate is determined by the binding energy of a molecule on the surface, referred to as a descriptor.^16^ The catalytic material at the peak of the Sabatier volcano achieved the maximum possible catalytic rate.

Catalysts that are stimulated to periodically change at frequencies comparable to the turnover frequency of surface reactions can overcome the limitations imposed by linear scaling relationships. As depicted in Figure 1h, the oscillation of the binding energy of adsorbates manifests itself as a horizontal shift on the Sabatier volcano plot. For sufficiently high applied catalyst oscillation frequencies, the time-averaged catalytic rate exceeds the Sabatier maximum up to the “resonant frequency” (purple line), defined as the applied frequency leading to a maximum in both catalytic rate and efficiency.^17,18^ Dynamic stimulation of catalysts also provides the capability to drive reactions away from equilibrium to a new steady-state conversion, either greater than or less than equilibrium defined by reaction conditions.^19^ This occurs via a catalytic ratchet mechanism, which imposes a kinetic bias on each elementary step reaction via energy input from light, strain, or applied charge. Finally, these new capabilities provided by a dynamic catalyst introduce new mechanisms to substantially improve the selectivity of products in complex multistep catalytic mechanisms.^20^

In this work, we present the current state of the art in stimulated dynamic and programmable catalysis and outline future opportunities and directions for the field. We begin by discussing modeling approaches used to understand and predict dynamic catalytic behavior and discuss which theories and models require further development. Next, we explore how different dynamic stimuli, such as charge, light excitation, temperature, and mechanical strain, influence catalytic performance (e.g., turnover, selectivity, stability) through distinct physical mechanisms. We focus on understanding their mechanisms and their challenges and limitations. We then examine the integration of dynamic stimulation with operando spectroscopy, coining a new philosophy of *“stimulando”* spectroscopy and characterization, and highlighting methods capable of detecting transient species, tracking energy flow, and resolving reaction pathways at (ultra)­fast time scales. Building on this, we propose potential reaction systems that could serve as standardized testbeds for benchmarking progress and comparing different stimulations. We conclude with an outlook on how dynamic catalysis could transform catalysis for sustainability.

## 2 Modeling of Stimulated Dynamic Catalysis

The description of catalytic reactions using physics-based models is challenging due to the large number of atoms and reaction phenomena that are often required to accurately represent the reaction system (e.g., a metal surface or a zeolite cavity). The development of density functional theory (DFT) has enabled faster computation of surface reactions, expanding our fundamental mechanistic understanding of catalytic reactions.^4,21^ It is common even for static catalytic reactions for the complexity associated with solvation effects,^22–24^ lateral interactions,^25^ and surface reconstruction^26^ to be omitted to limit computational demand and reduce the number of defined model parameters. The modeling challenge worsens when describing reactions where the rate-controlling step is given by a dissociative chemisorption reaction.^27^ This has been shown to be relevant for key reactions in the energy transition and food supply, such as methane steam reforming^28^ and ammonia synthesis.^29^ Microkinetic modeling of static catalytic reactions, despite several decades of development, remains a frontier challenge to achieve accurate prediction of rate and selectivity relative to experiment.

Like modeling of conventional catalysis, programmable catalyst modeling is inherently a multiscale problem, which now extends to the effect of stimuli at various lengths and time scales. This requires: (1) understanding the fundamental relationship between catalyst stimuli and surface–adsorbate interactions at the atomic scale, (2) capturing the influence of these stimuli on the microkinetic model, and (3) including stimuli-driven effects at the continuum scale (e.g., heat transport, transport of reactants and products to and from the catalyst). In this section, we focus on the implications of dynamic catalysis for the modeling of reaction microkinetics. The parallel development of catalytic resonance theory provides a framework for extending microkinetic models toward dynamic catalysis, which can be compared to real dynamic surface chemistries observed in experiments. It is essential to continue developing modeling methodologies that can accurately define the dynamic free energy landscape under (multiple) catalytic stimuli, starting from the atomic scale and ideally relying on accurate electronic structure calculations.

### 2.1 Microkinetic Models Including Dynamic Stimuli

Microkinetic modeling is at the core of theoretical modeling of catalysis. Dynamic catalytic reactions, characterized by periodic external stimuli such as light, strain, or electric fields, pose unique challenges in microkinetic modeling due to the complex interplay of reaction networks. A reaction network is a collection of elementary reaction steps transforming the reactants into (by-)products. Each step is associated with rate constants as a function of the applied stimulus. Without accounting for microscopic details of how a stimulus affects the catalyst or the adsorbates, the impact on the rate constants connected with each elementary step can be formally included in a coarse-grained manner by introducing time-dependent rate constants:

1 $$k_{i}\left(t\right)=A_{i}\left(t\right)e^{-\frac{E_{i}\left(t\right)}{RT\left(t\right)}}=\frac{k_{B}T\left(t\right)}{h}e^{-\frac{\Delta G_{i}^{‡}\left(t\right)}{RT\left(t\right)}}$$

Figure 2e provides an overview of the range of time scales associated with different stimuli. Certain stimuli result in a change of the position and/or energy of the transition state, such as strain or electrochemical potential (Figure 2a,c), which is reflected by the pre-exponential factor (*A_i_* (*t*)) as well as the activation barrier (*E_i_* (*t*)). Heating of the systems (Figure 2d) is described by a time-dependent temperature (*T*(*t*)). The influence of the stimuli can also be expressed via the Gibbs free energy of activation $\left(\Delta G_{i}^{‡}\left(t\right)\right)$ . As a consequence, the surface coverages θ*_i_* (*t*) of the different intermediate species can fluctuate, resulting in a change of the actual reaction rate, e.g.:

2 $$r_{i}\left(t\right)=-\theta_{i}\left(t\right)\cdot k_{i}\left(t\right)$$

**Figure 2:**
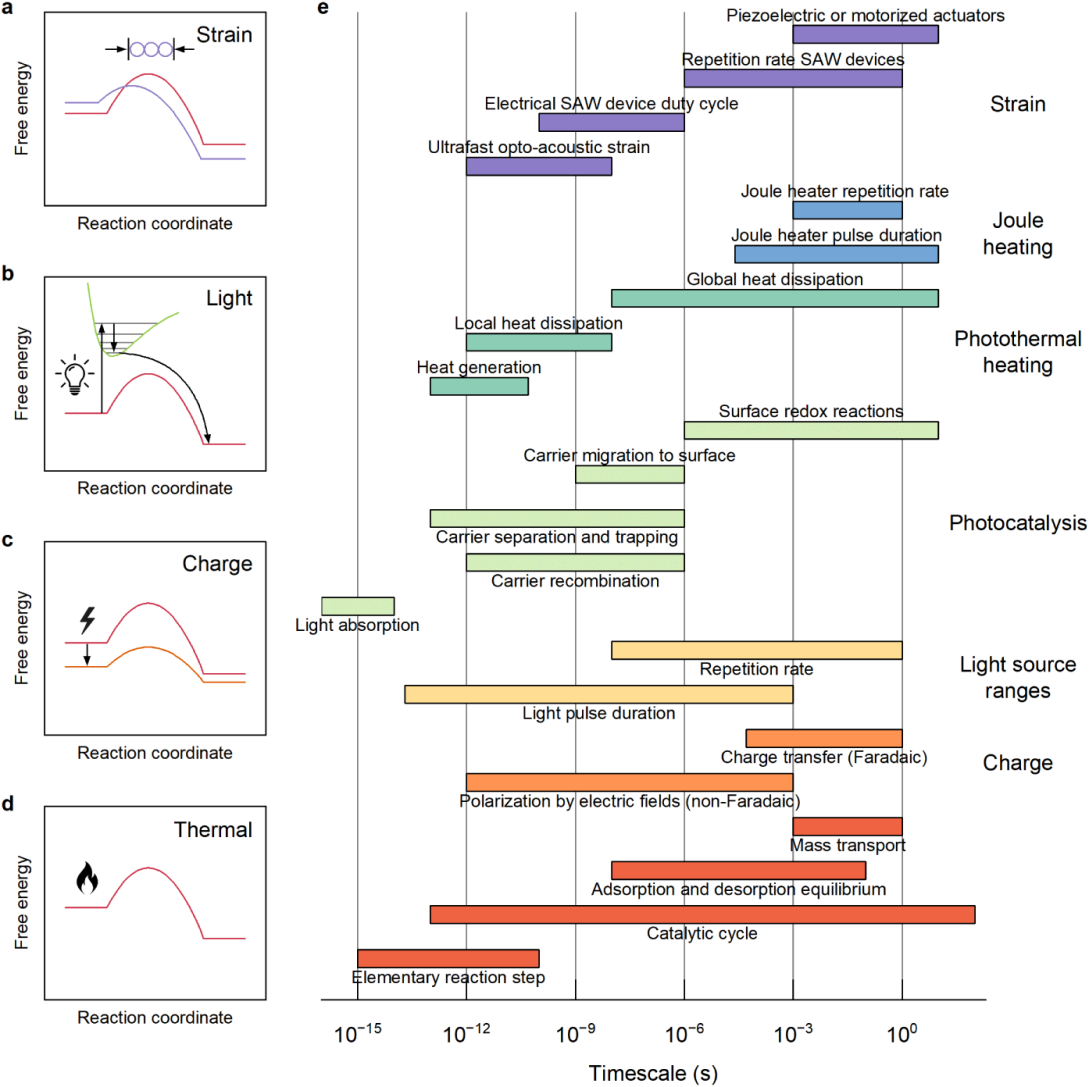
Schematic illustration of the effects of stimuli on minimum energy pathways and their relevant time scales. Modeling of conventional (heterogeneous) catalysis is based on the reactant-to-product free energy pathway defined by a system-specific reaction coordinate with the lowest energy barrier (d). Strain yields a direct modification of this path that can change both the position and the height of the barrier (a). Excitation by light (b) and charges in the form of the non-Faradaic and Faradaic processes (c) drive the system away from its electronic ground state and thus pose additional challenges for modeling. (e) Relevant time scales of heterogeneous catalysis (red, bottom) and the time scales of the various dynamic stimuli discussed in this work.

Other stimuli, such as light excitation (Figure 2b) can lead to multiple intersections between potential energy surfaces (PESs), significantly increasing the number of possible reaction pathways. Multiple PESs complicate the identification of a single rate-determining step, as periodic stimuli can dynamically shift the dominant reaction mechanism. While steady-state sensitivity analyses can guide the selection of key processes for small perturbations, these are limited in their applicability under dynamic conditions.

Microkinetic models use mean-field approximations, assuming perfect mixing and uniform access of reactants to active sites. However, this assumption breaks down in more realistic systems with transport limitations or dynamic modulation of active sites. For example, reactants might experience uneven access to catalytic sites, particularly when the reaction environment undergoes periodic structural or energetic changes. In such cases, kinetic Monte Carlo (KMC) simulations provide a more accurate approach by explicitly accounting for spatial and temporal effects, such as slow surface diffusion and local transport phenomena.^30,31^ Finally, understanding single excitation events, such as those induced by photons or electrons, requires the statistical treatment of reaction kinetics to predict the probabilistic outcomes of these discrete occurrences. Together, these approaches deepen our understanding of dynamic reaction systems and enable the design of more effective catalytic processes.

### 2.2 Catalytic Resonance Theory

While computational methods and molecular dynamics provide mechanistic insight into the molecular behavior of reacting surface species under stimulus, the emergence of forced-dynamic catalysts under light, strain, or charge/field also requires a fundamental understanding of the new kinetic behaviors arising from oscillating free energy pathways. Simulation of model dynamic reactions (e.g., A­(g) to B­(g) via intermediates A* and B*) has identified new kinetic mechanisms that can potentially enhance the rate of catalytic reaction, alter the steady-state conversion of reaction away from equilibrium, and drive reactions more selectively to targeted products. Under dynamic operation, it is predicted that programmable catalysts can accelerate reactions to a maximum catalytic rate above the Sabatier limit at the “resonance frequency,” which was defined as a maximum in the effective catalytic rate occurring when the applied catalyst oscillation frequency matches the rate-limiting kinetics of the overall catalytic mechanism.^17,32^ Identification of the oscillation amplitude and frequency of dynamic catalytic reactions requires a kinetic description of the elementary surface reactions, which define the Sabatier volcano; extension of the sides of the Sabatier volcano above the Sabatier limit defines the dynamic rates accessible by each rate-limiting step in the sequence of elementary steps of reaction.^6,19^ Application of stimuli to a catalyst surface at the reaction resonance frequency has the potential to accelerate reactions orders of magnitude beyond conventional static catalytic materials, providing a strategy for achieving faster catalysts with higher reactor throughput.

In addition to rate acceleration, the theory predicts that “energy ratchet” mechanisms involving dynamic free energy surfaces can arise from stimulation of the catalyst surface.^7^ For specific simulation conditions, the energy ratchet mechanism results in the utility of the stimulus energy input to drive reactions to extents of conversion different from equilibrium.^7^ This predicted capability of dynamic catalysts could enable the decoupling of reaction conditions from the reactor outlet composition. While catalytic reactors conventionally use temperature, pressure, and composition conditions to achieve a specific equilibrium composition, the use of dynamic catalysts that drive reactions to nonequilibrium product compositions would permit a broader range of operating conditions. This is particularly useful in reactions at extreme operating conditions, such as steam reforming of methane^33^ or the synthesis of ammonia.^8^ These methods of rate acceleration and nonequilibrium steady-state conversion promise to enable additional capabilities, such as the promotion of reaction pathways toward targeted products at high selectivity.^20^ Altogether, the fundamental mechanisms identified by these model systems provide a foundation for understanding more complex dynamic catalytic reactions and a strategy for improving the applied catalyst stimulus with dynamic kinetic modeling. Still, it remains to be scrutinized whether elementary processes at atomic length and time scales justify the intrinsic assumptions this modeling approach hinges on.

### 2.3 Challenges

The majority of the research conducted on these excitations assumes that the Brønsted–Evans–Polanyi (BEP) relationship holds, irrespective of the material, chemical environment, mode of excitation, amplitude of the stimulus, and frequency.^6,8,20,32,34–38^ The underlying assumption here is that the transition state energies are linearly correlated with the corresponding reaction energies. The BEP relationship has predicted the behavior of many traditional catalytic systems (without stimuli), which makes it an effective method for predicting the rates of elementary reactions and the ultimate turnover frequency of catalytic materials operating in the steady state.^39,40^ BEP relationships can be established from DFT calculations of a set of similar elementary reaction steps on different catalyst surfaces.

Determining linear relationships influenced by a catalytic stimulus is challenged by the need to describe the extent of the stimulus on the free energy of adsorbates. For instance, when using light excitation in plasmonic catalysis, hot charge carriers (i.e., electrons or holes) are generated, and they directly or indirectly interact with reactive species, subsequently leading to an increase in the product formation rate.^39,40^ However, other processes, such as thermalization of the hot charge carriers, compete with this process. Phonons in the catalyst may be directly transferred to the vibrational modes of the molecule.^41,42^ In this case, if the heat evolution is faster than the dissipation rate, the temperature can drastically increase, inducing surface reconstruction of the metal or even melting in the most extreme cases.^4,21^ These competing processes cannot be described by using a single atomistic model. Decoupling plasmonic and photothermal effects will be essential before establishing the relationships that enable the prediction of the overall reaction rate. In addition, the reliability of commonly made assumptions, such as the linearity of BEP relationships, should always be examined for each considered catalyst-chemistry-stimulus combination. As in static catalysis, the experimental results will need to guide the development of molecular models of the surface reaction kinetics.

Modeling stimuli-dependent reactions requires methods capable of describing transient states and kinetic variability, potentially revealing pathways that shift under varying degrees of stimulation to favor the formation of specific active sites with enhanced catalytic rates. The types of stimuli inducing the oscillations in dynamic catalysis widely vary, from electronic changes induced by localized electric fields in catalytic condensers to temperature changes caused by photothermal effects. This diversity of stimuli requires tailored theoretical models that accurately describe the reaction kinetics of the reactants and products. For instance, Wittreich et al.^8^ modeled the impact of lattice strain on a metal on the reaction free energy landscape by calculating the energetics of all the reaction intermediates involved in the production of ammonia from nitrogen and hydrogen at different extents of strain. This enabled the development of a kinetic model to estimate the influence of the dynamic strain frequency on the reaction rate. To describe charge and electric field effects on catalysis, electronically grand-canonical methods are available to calculate the electronic structure under controlled potential conditions, simulating the application of an external potential under electrochemical conditions.^43^ In sharp contrast, when the pulse involves the discharge of a plasma pulse, the complexity of the calculation drastically increases, making it difficult to generate general catalytic models that describe dynamic catalysis.

A model description of a dynamic catalytic reaction that continuously oscillates between electronic and physical configurations is expected to significantly increase the complexity of the catalytic model and the number of necessary parameters. Dynamic models utilize chemical descriptors, such as the binding energy of a specific adsorbate (e.g., binding energy of N*), that are related to a specific catalyst stimulus, such as strain. In the simplest case, a linear relationship between the stimulus, such as percent strain at the active site, and adsorbate binding energy introduces two new parameters (slope and offset). Every other adsorbate binding energy and transition state can then be related to the chemical descriptor, thereby introducing at least two new parameters (for linear relationships) each. The number of model parameters will continue to expand with the inclusion of additional complexity, such as nonlinear adsorbate–adsorbate or transition state scaling models. Due to this significant expansion in dynamic model parameters, the extent of uncertainty must be defined^44^ when discussing the expected rate enhancements or improved selectivity to products achieved by dynamic operation of the catalyst.

### 2.4 Outlook

Figure 3 summarizes the relative ease with which dynamical modeling can account for the effect of different stimuli. Molecular dynamics (MD) simulations are a tempting approach to investigate dynamic and resonant catalysis by capturing atomic-level details under time-dependent conditions. However, for MD simulations to account for the dynamic nature of the catalyst, the simulated catalyst area and time scales must be sufficiently long, i.e., reaching or ideally exceeding the time scales of stimuli as depicted in Figure 2. This requirement often exceeds length and time scales that can be reached with direct DFT-based molecular dynamics.^45,46^ Sometimes conventional interatomic potentials can help to overcome these limitations,^47,48^ but often an accurate description of the free energy surface is required that cannot be provided by such potentials. That is because they are based on simplified functional forms that often are not flexible enough to describe the complex set of interatomic interactions occurring in catalyst systems. For example, applying external pressure gives rise to distorted geometries with shortened bond distances that are typically not included in the parametrization procedures of such potentials.

**Figure 3:**
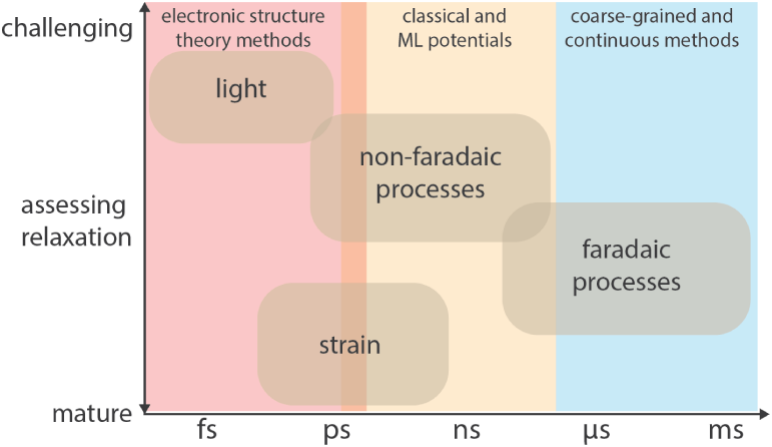
Overview of multiscale modeling methods for stimulated catalysis. Methods to describe the interaction of catalysts with different stimuli are organized schematically in terms of their maturity and time scale. External fields, such as electric potentials and mechanical strain, can be directly incorporated into molecular dynamic simulations, which can also be done in a dynamic or oscillatory way.^64–66^ External electric fields are routinely implemented to model electrocatalytic processes,^67–71^ and strain-induced effects can be modeled by deforming simulation cells.^72,73^ Light-induced dynamics and resonant effects (e.g., plasmonic excitation or vibrational resonances) are more challenging to simulate due to the quantum mechanical nature of photon-matter interactions. ML: machine learning.

Machine-learning interatomic potentials (MLIPs) can provide a computationally efficient means to achieve near-*ab initio* accuracy while significantly extending the accessible time and length scales^49–52^ and allow for the efficient evaluation of reaction rates for dissociative chemisorption reactions on surfaces.^53–55^ MLIPs are machine learning models, e.g., artificial neural networks or Gaussian process regression models, that are trained to reproduce results from accurate first-principles reference methods, such as DFT. These potentials are particularly useful for studying dynamic catalytic systems, where the catalyst undergoes structural changes or reaction environments fluctuate over time. Overall, MLIPs enable simulations that capture the interplay between dynamic catalyst surfaces and reactants at time scales unreachable by traditional DFT-based MD.^56–59^

Reaction mechanisms in dynamic and resonant catalysis can be highly complex, since the catalyst changes during the reaction. To explore reaction coordinates associated with dynamic and resonant catalysis, enhanced sampling methods are, therefore, invaluable.^60^ For example, metadynamics tracks collective variables (e.g., bond distances or angles) to bias the system along reaction pathways and intermediate states that might be inaccessible in conventional MD simulations or require exceedingly long simulation times.^61^ This enables, for instance, the study of systems where reaction dynamics are influenced by oscillatory or periodic modulation of external conditions.^62,63^

## 3 Charge and Current in Dynamic Catalysis

The ability to introduce specific amounts of energy is key to controlling the catalytic activity and reaction pathways. This section focuses on such “energy quanta” in the form of charge supplied by external sources, with some overlap with photoinduced charges and localized charge induced by local electric fields. With increasing access to solar and wind energy sources, the method of activating catalytic systems with renewable charge accumulation is becoming more attractive for driving industrial reactions. The goal is to improve the reaction rate and selectivity of targeted chemical and fuel products, leveraging the fast, controllable, and dynamic nature of charge. Charge modulation requires consideration with regard to kinetics, reaction thermodynamics, the nature of the electronic or electrochemical promotion, rate-limiting steps of specific reactions, and the structure of the catalyst and active site.

From a kinetics standpoint, charge activation can improve reaction rates by (1) reducing energy barriers related to bond breaking and formation, and (2) altering the adsorption and desorption of molecules on surfaces, thereby shifting species coverage and steering equilibrium toward specific products. An opportunity lies in the ability to control surface charge in a time-dependent manner (<milliseconds), enabling rapid modulation of the catalytic surface on the time scale of catalytic turnover frequencies. By modulating the catalytic surfaces between electronic states, it becomes possible to access new reaction pathways or accelerate otherwise slow reaction steps. This opens avenues to explore unconventional catalyst compositions and charge profiles that can influence reaction rates, leading to better catalytic processes.

From a thermodynamic standpoint, introducing charge to the catalytic surface alters the electronic properties of active sites, such as the Fermi level, density of states, and band bending at the catalyst surface. This in turn shifts the enthalpy and entropy of adsorption of surface species and thus the overall Gibbs free energy of the reaction, altering the favorability of competing reaction pathways. Enthalpic changes typically pertain to the strength of the interaction between the surface and chemical species, while ordering and mixing of adsorbates on the surface contribute to the entropic changes of the system that together determine the reaction equilibrium.

The methods to introduce charge to a catalyst can be classified into two broad categories, which are Faradaic and non-Faradaic processes. Faradaic processes involve direct electron transfer between the catalyst and the chemical species through redox events, where positive or negative charges are constantly exchanged to drive chemical transformations. These reactions are common in electrochemical cells, where applied potentials generate a flow of electrons that directly affects the reaction kinetics. Moreover, variation in the electron energy shifts the overall thermodynamics of the electrochemical half-reactions. Common examples are water splitting, the electrocatalytic reduction of CO_2_,^74^ electrochemical ammonia synthesis,^75^ and anodic oxidation in organic electrochemistry,^76^ where electrons supplied by the electrode participate in bond-breaking and bond-forming steps. In each case, the catalyst undergoes changes in the oxidation state, altering the energy of transferred electrons and accelerating a specific redox path for the desired transformation.

In contrast, non-Faradaic charge modulation of catalysts does not involve net charge transfer to chemical species but instead influences catalysis through modulation of the Fermi level at the active site or local variation of the electric field at the surface (Figure 4a). These effects can reorganize surface atoms, alter the adsorbate binding strength, modulate adsorbate molecular orientation (Figure 4b), or generate defects, all of which alter the catalytic properties of the active site. Examples include the NEMCA effect (Non-Faradaic Electrochemical Modification of Catalytic Activity), where ionic or electronic polarization across a solid electrolyte accumulates ions near the catalytic active site.^77^ Ferroelectric and piezoelectric materials are two other examples that use surface polarization under electric fields or mechanical strain to modulate electronic surface properties.^78,79^ Magnetoelectric coupling and electrostatic charging, such as in the case of the catalytic condenser, also enable precise control of surface energetics without altering the net oxidation states of active sites (Figure 4c).^11,80^ These systems demonstrate that surface reactivity can be dynamically tuned through charge or field effects alone, offering a complementary and often more energy-efficient strategy compared to Faradaic electrocatalytic processes.

**Figure 4:**
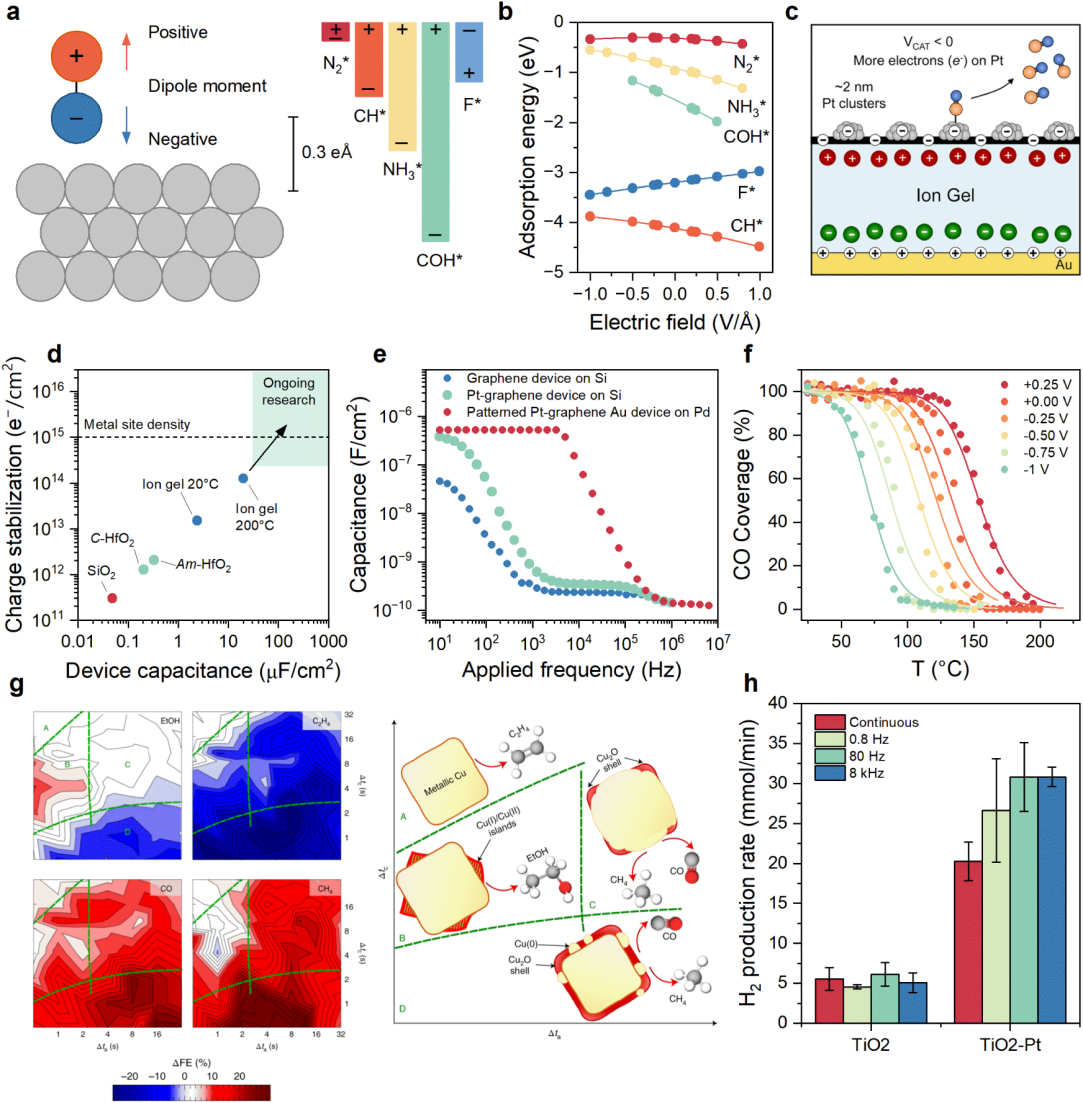
Dynamic catalysis modulated by charges and electric fields. (a) Cartoon of a surface dipole on a Pt(111) surface with the magnitude of selected surface dipoles (F*, CH*, COH*, NH_3_*, and N_2_*) shown on the right.^88^ (b) Variation of adsorption energy with varying electric field for adsorbates F*, CH*, COH*, NH_3_*, and N_2_* on the Pt(111) surface at the atop site.^88^ (c) Pt/C on an ion gel catalytic condenser with negative charge condensation, affecting CO desorption from Pt.^89^ (d) Device capacitance and charge stabilization for catalytic condensers based on different insulating dielectric layers (silica, amorphous and crystalline HfO_2_, and ion gel at room temperature and 200 °C) for an applied voltage of 1 V.^89^ (e) Capacitance of various condenser devices as a function of applied frequency.^12^ (f) Adsorption isobars of the normalized CO coverage on a Pt/C ion gel condenser measured as a function of temperature from −1.00 to +0.25 at 0.25 V increments. The experimental measurements (dots) were fitted with Langmuir isobars (lines).^89^ (g) Faradaic efficiency changes for EtOH (top left), C_2_H_4_ (top right), CO (bottom left), and CH_4_ (bottom right) during potential-pulsed CO_2_ electroreduction with respect to a static −1.0 V potential, with different lengths of anodic (*x*-axes, Δ*t*_a_) and cathodic pulses (*y*-axes, Δ*t*_c_).^90^ Regions A–D correspond to regions where the catalyst has different structures and surface compositions, based on XAS and XRD data, as schematically depicted on the right. (h) Hydrogen evolution rate under continuous and periodic UV-LED illumination (365 nm) for TiO_2_ and Pt-decorated TiO_2_.^91^ Panels (a) and (b) were adapted with permission from Shetty et al.^88^ Copyright 2020 American Chemical Society. Panels (c), (d), and (f) were adapted with permission from Onn et al.^89^ Copyright 2024 American Chemical Society. Panel (e) was adapted from Onn et al.^12^ Copyright 2022 American Chemical Society. Panel (g) was adapted from Timoshenko et al.,^90^ Copyright 2022 Springer Nature. Panel (h) was adapted from Sordello et al.,^91^ Copyright 2021 American Chemical Society.

Both underlying mechanisms (Faradaic and non-Faradaic reactions) are similar, but a key difference between the two processes is the time scales at the charged surface interface (Figure 2e). Faradaic processes require charge transfer through redox events and are limited by electron and ion transport, double-layer charging, and often by the diffusion of reactants to and from the electrode surface in a liquid. These steps operate on time scales of milliseconds or longer, depending on the size of the system and electrolyte environment.^81^ As a result, the activation time for Faradaic modulation tends to be slower and is suited for dynamic operation in the >ms regime. In contrast, non-Faradaic processes, where surfaces are activated by fields or local polarization, respond at millisecond or faster time scales.^82^ Since these mechanisms do not rely on transport of chemical species or changes in oxidation states, they can occur within picoseconds to milliseconds, opening up interesting new possibilities for faster modulation in dynamic catalysis.

The ability to induce dynamic charging depends on catalyst design and material selection to support electron transport and integration of components such as dielectric films, membranes, and stack devices that can operate at high frequencies. Several systems with potential for dynamic catalysis have already been demonstrated in this context, including catalytic condensers,^11,12,83^ ferroelectric surfaces,^37,84,85^ gated transistors,^86^ and proton-exchange membrane systems.^87^ These dynamic catalytic technologies can change between positive and negative voltages, enabling transitions between catalytic states at speeds comparable to or faster than the turnover frequency of catalytic chemistry (>1 Hz). However, implementing such systems has considerable challenges related to reactor design, delivery of charge, scalability, and direct measurements of charge condensation, all of which will require advances in the coming decade to achieve utility for catalytic control. This section will describe the tunability of charge modulation, highlight potential catalytic platforms for controlling charge, discuss challenges, and provide recommendations for advancing the field of dynamic catalysis.

### 3.1 Tuning of Surface Charges and Limitations

Surface charges modulated by external stimuli (e.g., applied voltage, applied strain) create a tunable energy landscape unique to each stimulus type and extent. In electrocatalytic electrodes or dielectric film catalytic stack devices, the surface charge can be controlled by applying a voltage or electric field through an external power source. In electrochemical systems, the extent of charge accumulation at the electrode is governed by the formation of an electrochemical double layer. Typical field strengths are on the order of 10^9^ V/m, with surface charge densities of up to 1 C/m^2^ (∼10^14^–10^13^ electrons/cm^2^).^92,93^ In capacitive systems, the extent of charge modulation is limited by the thickness and dielectric strength of the insulating material (Figure 4d). Only specific materials, such as high-permittivity perovskites with dielectric constants exceeding 1000, can sustain such high field strengths and the associated large extent of charge condensation without incurring a dielectric breakdown.^94^ The speeds of response in both cases are constrained by the RC time constant of the circuit, setting limits on how fast the catalytic system can be modulated (Figure 4e).

Other related methods of external introduction of charge to surfaces include photoexcitation, where pulsed light generates transient surface charge variation with time constants between 10^–1^ and 10^–7^ s and typical charge densities below 10^–3^ C/m^2^.^95,96^ This method represents one of the more accessible ways to achieve high-frequency charge modulation. Within the same category, plasmonic materials can produce comparable charge densities through optical excitation, although the lifetime of these carriers is limited to femtoseconds unless efficiently harvested.^97,98^ Mechanical methods such as triboelectric generators, which operate through contact and separation, also produce comparable surface charge.^99^ In contrast, piezoelectric materials can achieve much higher surface charge densities, up to 1 C/m^2^, through applied mechanical strain, as detailed in the section on strain-induced effects.^100^ Additional material-stimulus combinations include thermoelectric materials subjected to pulsed heat sources (see Section 6 on Thermal Effects), ionized gases under alternating electric fields (plasma), and surfaces modified with functional groups that respond to chemical changes. In these systems, the upper limit of charge condensation and the achievable modulation frequencies remain poorly characterized, highlighting the need for further evaluation.

In practical applications, surface charge can be either generated directly within a catalyst material or induced externally. In the former case, the catalyst itself serves as a stimulus-responsive material. In the latter approach, nanoscale catalysts are deposited onto a stimulus-active material, as demonstrated for the concept of the catalytic condensers, where charge densities up to 10^14^ electrons/cm^2^ have been reported, with operational frequencies up to ≈10^4^ Hz (Figure 4c–e).^11,83,89^ Crucially, such catalytic condenser devices can modulate the binding strength of adsorbates by 20 kJ/mol when switched between 0.25 and −1 V potentials (Figure 4f), which brings programmable catalytic condenser devices closer to application. Furthermore, strained piezoelectric materials with integrated electrodes have been successfully employed for electrocatalytic water splitting at frequencies up to 20 Hz.^101^ The possibility of combining stimulus-active materials with a catalyst provides a huge space for future studies.

Quantifying surface charges or potentials is vital to assess dynamic catalysts, and both macroscopic and microscopic techniques exist to probe the electronic properties of surfaces. Macroscopic methods include the Kelvin probe, transient surface photovoltage, and photocurrent techniques,^95,102,103^ as well as electrochemical methods such as stepped or pulsed potentiometry and electrochemical impedance spectroscopy.^104,105^ Chemical field-effect transistors have been used for charge sensing in solid-state systems,^106^ and electrostatic and electrokinetic methods provide insight into charge distribution and mobility for colloidal suspensions and aerosols.^98,106–108^ Spectroscopic methods such as vibrational Stark shift spectroscopy,^92^ surface plasmon resonance,^109^ and second harmonic generation^98^ can probe interfacial electric fields and charge effects. At the microscopic level, scanning probe techniques like atomic force microscopy (AFM) and Kelvin probe force microscopy (KPFM) allow for spatially resolved operando analysis of surface potentials and charge distributions.^106,110,111^

While these techniques can accurately probe surface charges at the atomic scale, their application in assessing dynamic catalytic systems remains limited, particularly in terms of temporal resolution. Most of the techniques mentioned above do not resolve transient events associated with ultrafast charge modulation. Kelvin probes, for example, have frequency limitations below the MHz range and may only resolve changes on millisecond time scales. Similarly, while scanning probe techniques provide high spatial resolution, their temporal resolution is insufficient to monitor the submicrosecond time scale. Spectroscopic approaches like surface plasmon resonance or second harmonic generation can provide faster measurements, but they often require specialized setups and complex experimental environments that deviate from standard catalytic conditions.

Achieving high-resolution, ultrafast time-resolved measurements of dynamic catalyst behavior remains a significant experimental challenge. As dynamic catalysis continues to grow, there exists a significant need to develop more advanced characterization tools that match the temporal and charge-varying properties of time-varying surface chemistry. These tools will be necessary to provide insights into the mechanisms of fast switching that may influence catalytic reactivity and selectivity, paving the way for future developments in dynamic catalysis through a detailed understanding of dynamic surface chemistry.

### 3.2 Charges from Biases and Currents

Dynamic charge modulation has been explored in electrochemical approaches, particularly Faradaic-reaction systems, and their adaptation to catalytic reactions driven by thermal energy proceeds with only limited fundamental understanding. Faradaic catalytic reactions, driven by potential-induced charge transfer, are inherently suited for dynamic operation up to a limit of applied frequencies as determined by the inherent time constants of voltage modulation. Techniques such as pulsed or oscillating potentials, characterized by frequency, amplitude, and duty cycle, have been applied to various reactions, including water splitting, CO_2_ reduction, organic electrosynthesis, oxygen reduction, nitrogen fixation, and contaminant removal.^112–118^ These approaches represent a new catalytic paradigm that can improve reaction rates, selectivity, catalyst stability, and possibly energy efficiency.

Dynamic promotion of catalytic rates arises from two key factors. The first is surface dynamics, where oscillating potentials can restructure surfaces and adjust the coverage of the adsorbed species. For instance, XANES studies have shown that applying oscillating potentials can shift the oxidation state of copper, altering product selectivity in CO_2_ reduction reactions (Figure 4g).^90^ Similarly, in the electrochemical production of H_2_O_2_, in situ Raman spectroscopy revealed enhanced *O_2_^–^ and *OOH species on the electrode surface during the pulsed potential application. This was attributed to a synergistic effect between Li^+^ ions and transient electric fields, leading to a reduction in the reaction energy barrier.^119^

The second factor is modulation of the electric double layer (EDL) and associated mass transport near the catalyst surface. In steady-state electrocatalytic systems, the EDL stabilizes as electric field gradients and ion concentrations reach equilibrium.^120–122^ Dynamic modulation disrupts this equilibrium, leading to changes in the field polarization and local ion distributions near the surface. This periodic disturbance facilitates improved reactant access and product desorption.^123–125^ Under favorable conditions, it can reduce mass transport limitations, prevent over-reduction or overoxidation of intermediates, and help regenerate active sites.^126,127^

A growing area of interest now is the adoption of this dynamic approach to reactions that have relied on thermal-based activation. Early studies include alternating current field-promoted CO oxidation on Ni plates (5× rate, 1966), NH_3_ synthesis over Fe (16× rate, 1970), and C_2_H_4_ hydrogenation over ZnO (rate tuning via reaction order/activation energy, 1975) in fixed beds.^128–130^ Despite these early efforts, the lack of mechanistic insights into the dynamic catalytic mechanism has limited progress. A resurgence came in 2021 when Lim and coworkers applied oscillating potential to C_2_H_4_ hydrogenation over Pd/C using a three-electrode single-cell configuration.^131^ While TOF displayed a classic volcano-type dependence on static potential, dynamic potentials boosted TOF by up to ∼5-fold over the static peak. Importantly, this occurred under non-Faradaic conditions (Faradaic efficiency >2000%), implicating dynamic surface restructuring (e.g., periodic coadsorption/cleaning of C_2_H_4_ and H_2_) rather than direct electron transfer. However, not all dynamic conditions were beneficial; mismatched conditions between oscillation and surface kinetics led to suppressed activity in some cases.

### 3.3 Charges from Photoexcitation

In addition to introducing charge via an external bias, the standard approach in electrochemistry, the potential of a catalyst can also be controlled by light. Many photocatalysts consist of nanoparticles. Their small size leads to an equally small electrical capacitance.^132–134^ Adding or removing a few charge carriers to or from a nanoparticle results in a comparatively large shift in the Fermi level.^135,136^ When nanoparticles are used to catalyze a redox reaction, an asymmetry in the rates of oxidation and reduction can lead to an effective charging of the particle.^137–143^ The associated shift in the Fermi level might influence both the thermodynamics of the reaction and the binding energy between the catalyst and the reactive adsorbate.^143,144^

In photocatalyzed redox reactions, the absorption of light generates excited electrons or holes in the photocatalyst. These have a higher reduction (electrons) or oxidation (holes) potential compared to the nonexcited charge carriers. The driving force of the redox reaction is given by the difference between the increased quasi-Fermi level of the excited charge carriers and the reduction or oxidation potential of the adsorbates.^134^ As the quasi-Fermi levels not only reflect the energy of the individual charge carriers but also their numbers, the reactivity of the catalysts can be modulated by the light intensity.^134,136,142^ In the dark, the quasi-Fermi levels recede to the original Fermi level of the catalyst material without excitation.

Charging of nanoparticles during photoredox reactions was first discussed in the context of semiconductor and semiconductor-metal nanoparticles.^139,140^ For the latter case, it was demonstrated that charge accumulation exerts a significant influence on the charge carrier transfer between semiconductor and metal.^138,144^ Furthermore, for metal nanoparticles, a direct influence on the activation energy of redox reactions by charging the particles has been demonstrated.^136,145,146^ Notably, the group of Jain demonstrated that by carefully adjusting the particles’ Fermi level through charging, reactions that are otherwise thermodynamically unfavorable, such as the reduction of CO_2_, can be accomplished.^145,146^

Since light can be periodically modulated in a relatively simple way, we envision realizing the concept of resonant catalysis through modulating the charge of nanoparticles via controlled periodic illumination (CPI). By periodically shifting the Fermi level, the catalyst is intended to switch between a state with a high affinity for activating the reactants and a state with a low binding energy for the products.^143^ Initial attempts to implement resonant catalysis with CPI reported a promising enhancement of the photoefficiency for the degradation of formate anions by TiO_2_ aqueous slurries.^147,148^ Unfortunately, later studies could not confirm the enhanced reactivity, leading to the assumption that the alleged enhancement was the result of an inadequate experimental procedure.^143,149–152^ We speculate that one reason for these negative results is related to the presence of oxygen in the TiO_2_ slurries. Oxygen is known to be an efficient electron scavenger that prevents the buildup of a negative charge on the TiO_2_ particles and therefore the modulation of the Fermi level.^139^

Recently, a group of scientists around Pellegrino and Maurino, investigated the influence of CPI on the photocatalyzed hydrogen evolution reaction (HER) using platinum-decorated TiO_2_ particles.^143,149–152^ They reported a 50% increase in efficiency for CPI with modulation frequencies above 80 Hz (Figure 4h).^91^ More importantly, they demonstrated the presence of oscillations in the potential of the catalyst, which proves the periodic modulation of the particles’ Fermi level.^153,154^ We regard this investigation as a promising first step in exploring the potential of dynamic catalysis by light-induced Fermi-level modulation. These positive results suggest a possible increase in activity in the investigation of CPI for photocatalyzed redox reactions. To begin with, many details of the influence of CPI on the HER are still to be clarified.^143^ This includes the question of whether the observed shift in the particles’ potential is indeed the cause of the enhanced efficiency. Next, CPI should be applied to other photocatalyzed redox reactions and catalysts in order to identify universal governing principles of photoredox enhancement by resonant catalysis. Also, the factors that determine the charging and discharging mechanisms must be understood in more detail. Initial investigations report a strong influence of the electrochemical double layer, including the ligand shell.^133,135^ Finally, it should be clarified whether and why the presence of a metal is necessary to implement resonant catalysis by photoinduced shifts of the electrochemical potential, as the success of the HER on Pt@TiO_2_ seems to suggest.

These examples showcase that there is still much to be explored regarding the underlying mechanisms and material requirements for charge-induced dynamic systems, which again points to the need for better toolkits to improve our understanding of surface charge modulation, electric field interactions, etc. Future work in this area will continue to evolve, and this represents an exciting frontier in catalysis.

### 3.4 Challenges and Outlook

The advance of charge-based dynamic catalysis is shaped by critical challenges in understanding oscillating surface chemistry and controlling performance. The first challenge is the lack of tools that can directly measure the ultrafast transient changes in the surface potential or charge under dynamic conditions. Current characterization techniques, such as Kelvin probes and spectroscopic systems, are limited in temporal resolution within the frequency range of interest for dynamic catalysis. These characterization methods operate at best on time scales of milliseconds, while transient changes in dynamic catalysis may occur on the microsecond scale or lower. In this context, current methods capture only time-averaged characteristics, and they thus suffer from the inability to characterize the precise details of the dynamically induced catalyst changes, limiting our ability to fully understand and optimize these processes.

Another challenge lies in distinguishing the effects of charge and field modulation from artifacts related to surface heating, mass transport, or other nondynamic influences.^155^ Establishing standardized benchmarks or probe reactions will ensure that observed changes are due to dynamic promotion of chemistry independent of experimental artifacts, to facilitate meaningful comparisons across different research efforts. Some possible examples, such as CO methanation (e.g., CO + H_2_ to CH_4_), alkyne/alkene hydrogenation, and CO to CO_2_ oxidation are ideal model reactions for benchmarking dynamic performance between laboratories and research methods, as they are well-studied and provide platforms for measuring changes in rate, selectivity, and activation energy under various modulation conditions, including frequency, amplitude, and waveform shape.

Material development remains another major challenge for dynamic catalysis, with key considerations related to charge delivery and surface charging and discharging at ultrafast time scales. Understanding these materials requires assessment of different photoactive systems, a balance of high dielectric strength and dielectric breakdown, fast charge or carrier mobility, and structural stability under charge modulation conditions. Future studies can evaluate promising candidates such as perovskites that can be ferroelectric, piezoelectric, or photoactive, as they have better charge capacity compared to conventional catalytic materials.^9,156,157^ In such materials, there is the consideration of oxygen vacancies, lattice mismatch with substrates, thermal stability, active phases, etc., given that perovskites are also used as oxide membranes in solid oxide fuel cells. Additionally, dynamic materials, such as strain-coupled piezoelectrics or photoactive semiconductors, hold promise as self-actuated charge modulators, potentially expanding the dynamic catalysis field into additional material classes.

In conclusion, although dynamic catalysis via charge modulation presents compelling opportunities, substantial progress in materials engineering, measurement capabilities, and system integration is essential. By addressing these challenges, we can realize programmable catalytic processes.

## 4 Light and Excited States in Dynamic Catalysis

The dynamic manipulation of fundamental catalytic properties on kinetically relevant time scales has been the goal of dynamic or resonant catalysis for the past several years. While theoretical studies of dynamic manipulation of adsorbate binding energies on time scales relevant to adsorption/desorption and reaction steps (10^3^–10^15^ s^–1^) have shown order-of-magnitude enhancements in reaction rates, experimental evidence of this resonance remains scarce.^17^ The grand challenge for stimulated dynamic and resonant catalysis is the ability to exert significant changes in a catalyst’s electronics in a fast and repeatable manner. Light is uniquely equipped to tackle this daunting challenge, but its versatility and limited understanding of its influence on catalysts underscore the complexity of the emerging field of dynamic photocatalysis.

Static, continuous wave illumination has been used extensively as a stimulus for chemical reactions via electronic or vibrational excitation of light-responsive molecules, catalysts, and supports to generate excited-state energy carriers such as electrons, holes, redox-active species, and phonons.^158–161^ However, only a handful of studies are available in which light is used as a stimulus for dynamic catalysis. To transition from static to dynamic, on the one hand, we must leverage the wealth of existing knowledge on how static light influences catalysis and, on the other hand, understand how pulsed light protocols can resonate with underlying kinetics. Since light couples to many different effects that are covered elsewhere in this perspective (charge, strain, heat, and photothermal effects), in this section we will discuss the benefits, challenges, and our perspective on future use of photons for dynamic catalysis.

### 4.1 Benefits and Success Stories

Light has many characteristics that make it particularly attractive as a stimulus for resonant catalysis. Light is tunable in wavelength, intensity, temporal coherence, phase, spectral bandwidth, and polarization. In the spatial domain, it is possible to manipulate beam size (down to the diffraction limit), divergence, and structure (e.g., speckles or grids), which enables spatial-selective excitation of catalysts. Furthermore, light can be modulated with an arbitrary waveform (enabled by the nanosecond resolution of electronics) or pulsed. Pulsed light sources are widely available, varying from the nano- to microsecond (LEDs) down to the femtosecond (lasers) time scale, and repetition rates variable from single shot to GHz.^162^ Both LEDs and lasers are stable and reliable light sources that can operate almost continuously for years with little to no maintenance in industrial settings such as laser processing of materials, micromachining of devices, and the production of LEDs. Overall, light is an extremely flexible and versatile stimulus that enables elaborate control over where, when, and how many excited states are produced in a catalytic material.

Some promising examples have already emerged. Recent studies have demonstrated that the photon-mediated desorption of surface-bound species can enhance catalytic performance by dynamically modulating adsorbate apparent binding energies. For instance, 440 nm light has been shown to drive CO desorption from Pt nanoparticles through nonthermal energy exchange, thereby boosting reaction rates in systems where CO desorption is rate-limiting.^163^ The specific excitation of the Pt-CO bond was shown to enhance the CO oxidation rate in hydrogen-rich streams.^163^ More strikingly, modulated illumination at the same wavelength at kilohertz frequencies has outperformed continuous-wave (CW) light in methanol decomposition, achieving higher quantum efficiency (Figure 5a).^13^ This enhancement (≈30% higher rate at 3.5 kHz repetition rate) was attributed to the oscillation between two different rate-determining regimes. In the dark, the product (CO) binds strongly to Pt, poisoning the surface and limiting methanol activation (Figure 5b). Under illumination, however, the rate is limited by the reactant activation.

**Figure 5:**
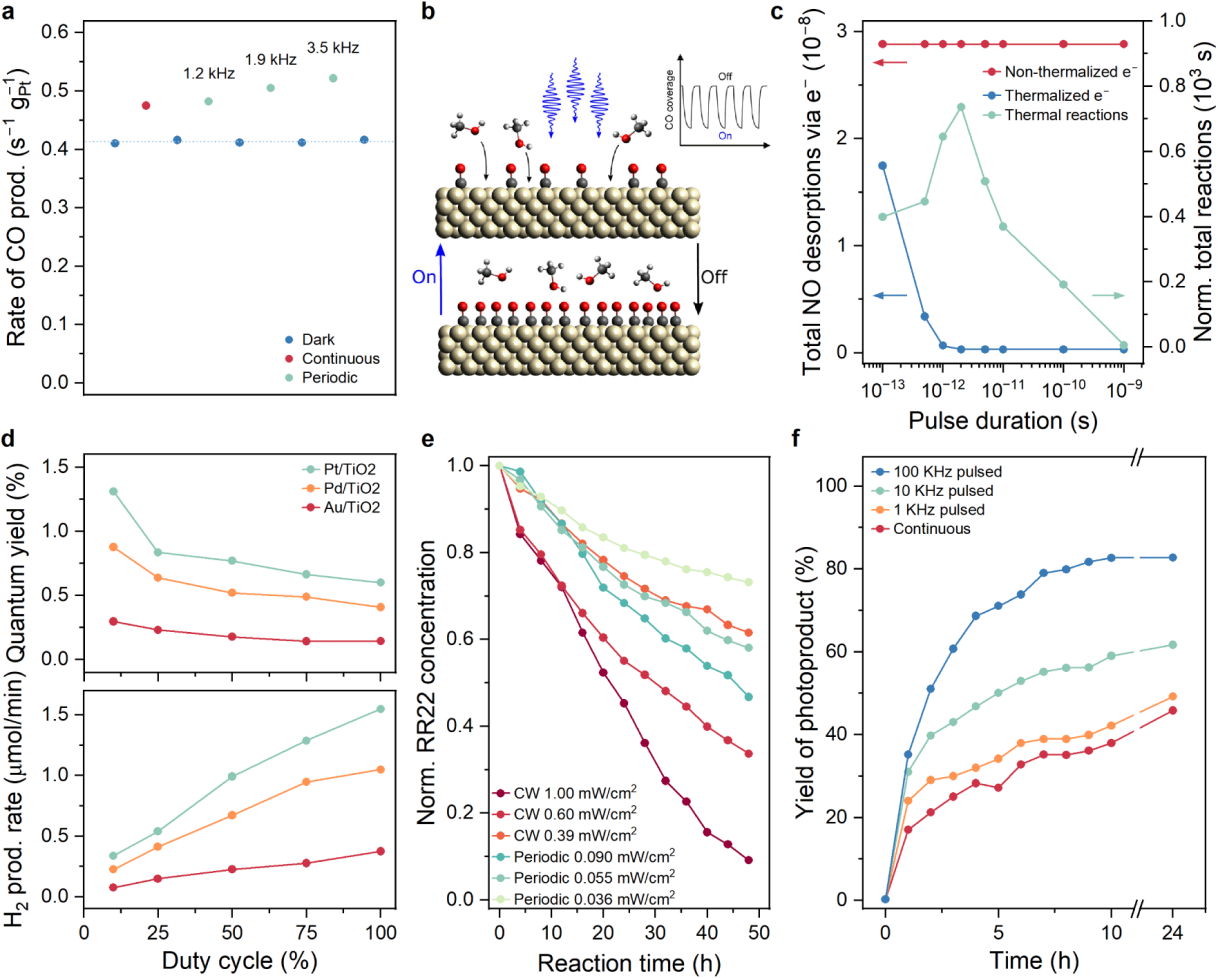
Examples of dynamic catalysis under periodic or pulsed illumination. (a) CO production rates from methanol decomposition on Pt/SiO_2_ under continuous and periodic (chopped) illumination at a fixed time-averaged intensity of 1.3 W/cm^2^ at 440 nm. (b) Cartoon illustrating how periodic light enhances methanol decomposition on Pt by alternating between CO-poisoned (dark) and clean (illuminated) states, boosting overall reaction rates beyond static conditions. Panels (a) and (b) were adapted with permission from Qi et al.^13^ Copyright 2020 American Chemical Society. (c) Theoretical total NO desorption events from a Pt bow-tie antenna due to nonthermal (red) and thermalized (blue) electrons as a function of pulse duration, with normalized Arrhenius-based thermal desorption (green) shown for comparison. Adapted with permission from Schirato et al.^164^ Copyright 2024 American Chemical Society. (d) H_2_ production rate (bottom) and quantum yield (top) from photocatalytic HCOOH decomposition over noble-metal-supported TiO_2_ photocatalysts at varying chopping duty cycles, at a frequency of 7.14 Hz, and at 0.25 W/cm^2^ lamp intensity (Xe lamp). Data extracted from Wong et al.^165^ (e) Photocatalytic degradation of dye “Reactive Red 22” on TiO_2_ at continuous (red-orange) and periodic UV-LED illumination (green, 10% duty cycle, 1 Hz). Data extracted from Wang and Ku.^166^ (f) Formation of Cu-complex photocatalyzed product under different blue LED irradiation modes at same intensity: pulsed (1–100 kHz, yellow/green/blue) and continuous (red), monitored by ^19^F NMR. Data extracted from Nicholls et al.^167^

In other work, modeling of ns–fs pulsed excitation of a plasmonic photocatalyst demonstrated how transient negative ion formation by hot (nonthermalized) carriers can result in the modulation of effective binding energies at ultrafast time scales (Figure 5c).^164^ Depending on the pulse duration and wavelength, the relative reaction rate contributions of lattice heat, nonthermalized carriers, and thermalized carriers could be modulated. These results facilitated the prediction that different experimental results may be obtained with fs-pulsed illumination than with ns-pulsed illumination, as well as differences in total reactivity due to enhanced heat localization for fs–ps pulsed illumination (further discussed in the Section 6 on heat). Taken altogether, these examples effectively support the idea that modulating binding energies through light-sensitive catalytic parameters on time scales faster than turnovers can enable unique reactivity.^13^

For heterogeneous catalysts in aqueous environments, favorable effects of pulsed illumination have been described since 1993,^147,165^ when it was first described how modulated excitation (72 ms on, 1.45 s off) increased the reaction yield of formate decomposition by 500%. Again, multiple intertwined mechanisms may play a role that is challenging to distinguish. A key effect is improvement of charge transfer kinetics. During the dark intervals of pulsed illumination, fewer photogenerated holes accumulate, reducing recombination and allowing more electrons to reach catalytic sites (e.g., Pt on TiO_2_) and drive reactions like hydrogen evolution, thereby boosting overall photocatalytic activity.^165^ This is corroborated by enhanced photocurrents and decreased charge-transfer resistance under pulsed light, particularly at low duty cycles, where the enhancement factor in photocurrent and quantum yield showed strong correlation (Figure 5d).^165^ Periodic dark phases may also prevent the accumulation of reactive intermediates that otherwise engage in nonproductive or recombination pathways, a mechanism first suggested by Sczechowski et al., who observed up to a 5-fold improvement in photoefficiency with carefully timed light and dark intervals.^147^ Moreover, dark periods allow surface regeneration, such as reactant adsorption or relaxation of surface states, aligning the light–dark cycling with the time scale of slower adsorption or reaction steps.^147,165^ For example, it was found that the quantum yield of dye degradation on TiO_2_ can be increased by a factor of 10 under pulsed UV-LED illumination (Figure 5e).^166^ In some systems, periodic illumination may also facilitate product desorption via dynamic shifts in surface potential, resembling the catalytic resonance effects observed in time-modulated catalysis.^165^ These enhancements, seen across Pt/TiO_2_, Pt/CdS, and Pt/C_3_N_4_ photocatalysts, suggest that pulsed light promotes a favorable balance between excitation and catalytic turnover, although the benefits are strongly dependent on the photophysical properties of the system and its rate-limiting steps.^165^

The use of pulsed LED light has also been explored for synthetic homogeneous photoredox catalysis.^167,168^ Importantly, it is shown that each system responds very differently, with some reactions being enhanced, others experiencing no difference, and some even being negatively influenced.^168^ This observation underlines that pulsed illumination does not automatically guarantee improvements. Although no clear evidence has been provided thus far, it was hypothesized that several interconnected mechanisms play a role. First, matching the pulse frequency to the excited-state lifetime of the photocatalyst may ensure that photons are delivered when the catalyst can most efficiently absorb them, as seen in studies where a 100 kHz frequency aligned with the 10 μs lifetime of a copper catalyst, leading to significantly improved yields (Figure 5f).^167^ Pulsing may also help mitigate catalyst and intermediate decomposition by introducing dark phases that allow these species to relax or react without continued irradiation. The approach may also avoid photon saturation, where excess continuous light no longer increases product yield and instead leads to energy waste and potential side reactions. In reactions involving radical chains, pulsed light may favor propagation over repeated initiation, improving the quantum efficiency. Moreover, by spacing out irradiation, pulsing can reduce competing photoreactions of intermediates, giving time for the desired light-independent steps to occur. In some cases, the higher peak intensity of light during each pulse may even facilitate otherwise inefficient multiphoton or nonlinear processes. The complexity and diversity of these mechanisms advocate for an improved understanding of and further experimentation to unlock the full potential of pulsed illumination in photoredox catalysis.

### 4.2 Challenges and Perspectives

Overall, dynamic photocatalysis presents a broad spectrum of variables and parameters that can be finely tuned to enhance the reaction efficiency and selectivity. However, this versatility introduces considerable challenges. One primary challenge is the coupling of chemical and physical processes occurring across vastly different time scales. For example, for heterogeneous photocatalysts, photoexcitation occurs within femtoseconds, charge carrier separation and trapping typically take nanoseconds, while surface redox reactions span from microseconds to seconds.^161,169^ Associated catalytic processes are, e.g., elementary reaction steps, the catalytic cycle, and mass transport, which occur at time scales of femto- to picoseconds, pico- to microseconds, and seconds, respectively.^170^ Multistep reaction kinetics are often limited by the slowest step, making it crucial to evaluate which processes are influenced by dynamic illumination in the context of the rate-determining step. Fortunately, tuning the repetition rate of the light source enables optimization of the catalytic efficiency by aligning excitation timing with specific reactive stages. Meanwhile, theoretical modeling plays a pivotal role in providing a comprehensive understanding of these processes and their interactions with light.

The practical delivery of light to a heterogeneous catalyst, especially in conventional powder samples, poses additional challenges. Scattering and minimal penetration through the catalyst bed often complicate quantification of absorbed, reflected, and transmitted photons, turning calculations of quantum efficiency into an optical puzzle. Ongoing development of improved photoreactors relying on light-coupling via optical fibers or porous light-guiding systems offers solutions for some of these limitations.^171–173^ However, challenges are further compounded in dynamic illumination, where the light intensity varies throughout the experiment. High power densities can compromise material integrity, while photonic quantum efficiencies (the number of reaction events per input photon) often saturate at elevated light intensities.^174,175^ Accurate reporting requires consideration of these effects, particularly at low duty cycles, where peak and average power densities may differ significantly.

Additionally, it is important to consider the fluence of the light delivered to the catalyst. Power density (W/cm^2^) or photon flux (photons/s) are often used to describe light sources, but in the context of photocatalysis it may be more useful to normalize photon flux to the number of active sites (photons/site/s). For example, a 440 nm laser with 1 W of optical power incident on a photocatalyst corresponds to a photon flux on the order of 10^18^ photons/s. Assuming the illuminated sample area is 1 cm^2^ and the active site density is 10^15^ sites/cm^2^, this equates to only ∼10^3^ photons/site/s. For comparison, the frequency of a reactant gas impinging on a catalytic surface is typically 10^5^–10^8^ molecules/site/s. This suggests that high photon fluxes may be necessary to exert appreciable changes in catalytic performance.

A consequence of the high power density, however, is significant photothermal heating. Accurate measurement of the temperature at the catalytic surface (rather than the bulk temperature) is nontrivial and becomes even more complicated under dynamic illumination. While macroscopic fluctuations can be controlled with careful thermocouple placement, thermal gradients may persist at the micro- or nanoscale, both temporally and spatially. Decoupling thermal from photochemical contributions to reaction rates therefore remains one of the grand challenges in dynamic photo­(−assisted) catalysis. More accurate measurement and control of photothermal contributions are helpful but difficult. One alternative approach is to rely on chemical signatures, such as changes in selectivity or reaction order upon illumination.

Lastly, designing reactors to benchmark dynamic photocatalysis across the community is critical for progress. Current reactors, such as the Harrick cell, allow for simultaneous in situ spectroscopic and reactivity measurements but have notable limitations, i.e., nonideal reactor behavior and two-dimensional illumination inducing photon gradients in the catalyst bed. Future efforts must focus on improving reactor designs to support standardization and reproducibility, ensuring that the field continues to advance toward realizing the full potential of dynamic catalysis. The ideal laboratory reactor to study the fundamentals of dynamic and stimulated photocatalysis would have a small volume and thin catalyst layer to allow for uniform light distribution throughout the catalyst and chemical analysis of small amounts of product on short time scales. Additionally, it needs an optical window that is compatible with many different wavelengths of light that can be used both to stimulate and to monitor the catalyst: UV, VIS, NIR, mid-IR, and X-ray, ideally in imaging mode. These all point to microfluidic flow reactors, for example, with ultrathin (∼20 nm) silicon nitride or silicon oxide windows. A number of companies sell these for in situ heating experiments in TEM, both in gas and liquid, but the same cell can also be used for optical stimulation and spectroscopy. In the short term, such microflow cells provide more detailed information about the underlying mechanisms in dynamic and resonant catalysis and also curtail mass transport limitations. For ultimate implementation, new larger-scale reactors need to be designed in such a way that they can provide optical access to a large catalyst surface area.

## 5 Strain in Dynamic Catalysis

Strain, defined as the displacement of atoms from their equilibrium position, induces changes in the electronic structure of the catalyst. As a result, the binding energies (BE) of reaction intermediates and transition states can be modulated (Figure 6). The utilization of lattice strain in order to alter the catalytic performance and the scaling relations of catalysts is well-established in technological and research fields, such as in fuel cells and bi- and multimetallic supported catalysts.^10,175–178^ Here, the equilibrium (surface) lattice parameters, and thus the associated strain of flat surfaces as well as nanoparticle systems, can be controlled by, e.g., chemical doping or epitaxial strain from the underlying substrates.

**Figure 6:**
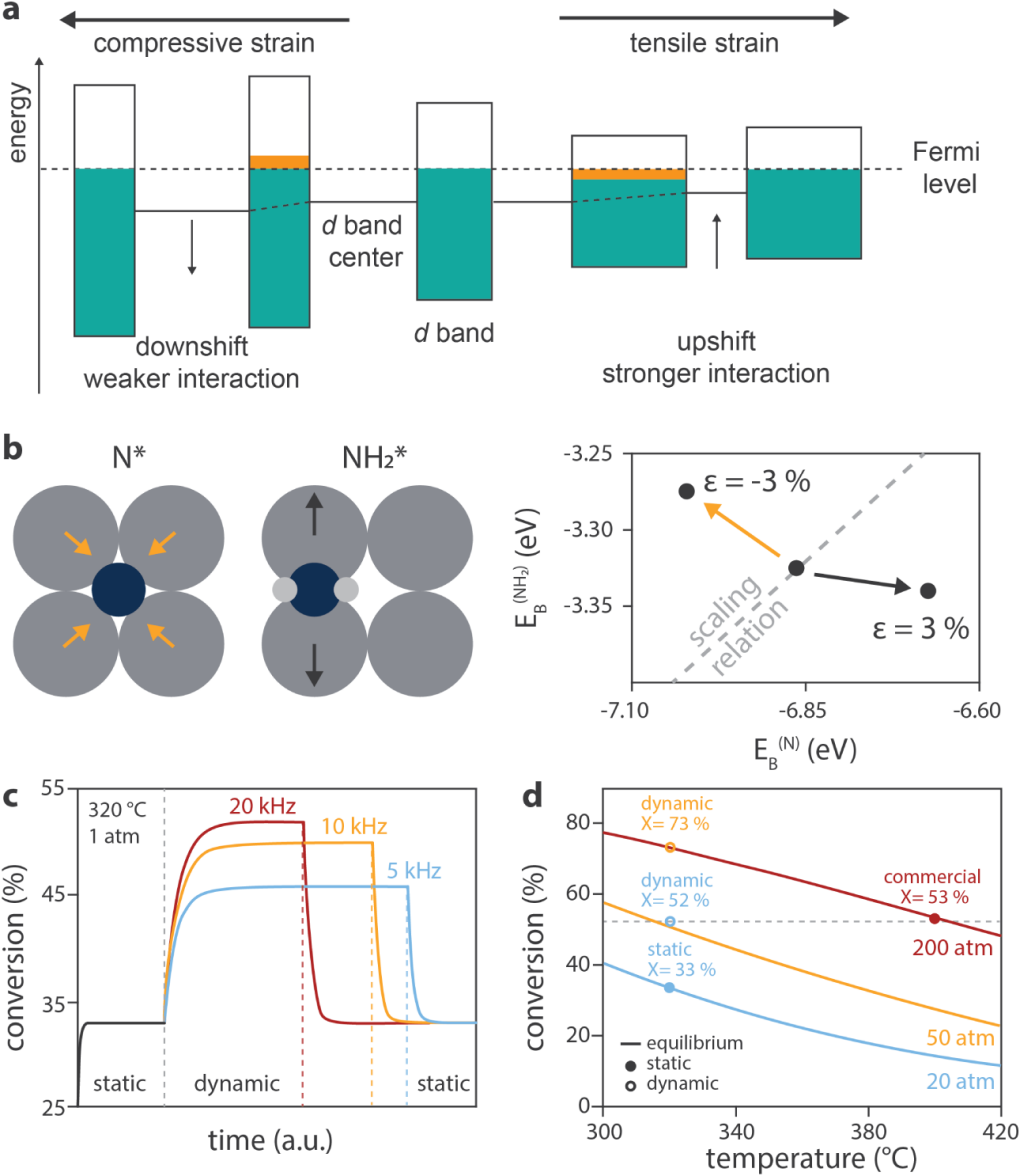
Fundamentals of strain effects in catalysis and their potential for dynamic catalysis. (a) Model to explain the electronic effect of compressive and tensile strain on the d-band center of transition metals.^184^ Tensile strain corresponds to more separated atoms and a decrease in atomic orbital overlap, leading to a decrease in d-bandwidth. Assuming no charge transfer, for late transition metals (d-band more than half-filled), the d-band must shift upward, toward the Fermi level, leading to stronger interactions with adsorbates. Conversely, compressive strain causes weakening of interactions with adsorbates (green: filled states; orange: filled or empty states out of equilibrium). (b) Eigenforce model: forces induced by NH_2_ and N surface atoms on undistorted surface atoms, showing the opposite effects of the two adsorbates on surface stress. When uniaxial strain is applied, the two adsorbates will be stabilized differently. Adapted with permission from Khorshidi et al.^10^ Copyright 2018 Springer Nature. (c) N_2_ conversion to NH_3_ calculated based on microkinetic simulations on a Ru nanoparticle, for the static case, and switching to dynamic stimulation by ±4% square wave strain at various frequencies. The stimulation promoted the conversion above the equilibrium conversion of 33% at 320 °C and 20 bar to a periodic solution as high as 52% at 20 kHz. (d) Comparison of ammonia synthesis activity with respect to equilibrium (lines) as a function of temperature at indicated pressures, for the static (●) and dynamic cases (○, 20 kHz). Adapted from Wittreich et al.^8^.

The order of magnitude of the effect of strain on the BE of adsorbates is tens of meV per percent strain.^179^ Compression tends to weaken binding, while tension tends to strengthen binding. In some cases, the variation of binding energy due to strain can be much larger: for example, for a carbon atom on a platinum (111) surface, the theoretical variation can be 0.6 eV over a 2% biaxial strain range. A number of rules of thumb are available to understand the susceptibility of catalysts to strain.^179^ Adsorption sites with higher coordination numbers tend to exhibit higher susceptibilities, and adsorbates with higher valency tend to be more susceptible. Among common adsorbates, N- and O-bound adsorbates tend to have higher susceptibilities, while among common close-packed surfaces, the susceptibility is typically ordered (Pt, Au) > Pd > (Ag, Cu).

The effect of strain on the BE of adsorbates can be explained by two models: the d-band center model (Figure 6a)^180,181^ and the eigenforce model (Figure 6b).^179,182,183^ According to the d-band center model, tensile strain should lead to a stronger BE for all adsorbates. This is the case for most adsorbates. Consequently, the change in BE induced by strain for different adsorbates is usually correlated, in line with the common scaling relations of catalysis. However, calculations suggest that some notable exceptions exist. These exceptions can be predicted based on the eigenforce model. Eigenforces are the forces that an adsorbate exerts on the substrate atoms upon adsorption. When eigenforces are relieved, for example, by strain, the adsorbate BE increases.

In most cases, eigenforces point outward from the adsorption site, so tensile strain leads to stronger bonding. However, in rare cases, such as for N* in a 4-fold site (Figure 6b), the eigenforces point inward (in which an adsorbate draws in the surrounding substrate atoms), resulting in weaker bonding under tensile strain. Such counter-directional differences in the strain response of BE can potentially be harnessed to strongly influence reaction rates, as they correspond to negative values of the parameter γ of resonance theory.^19,185^ In some cases, a counter-directional strain response can be directly engineered. For example,^10^ in the case of uniaxial strain, there will typically be a Poisson response in the orthogonal direction. In such a scenario, hollow sites such as 3- and 4-fold sites will tend to be under tension, while certain bridge sites will tend to be under compression locally. Thus, reactions that involve adjacent binding sites for adsorbates involved in the reaction sequence may have counter-directional effects.

While chemical approaches provide a ready means to synthesize a strained catalyst, the strain effect is typically mixed with a composition effect, as catalysts at different strain levels will have a different composition; this is generally referred to as the ligand effect.^186^ Furthermore, such approaches are not obviously amenable to dynamic operation at different strain levels at the time scales necessary for programmable and resonant catalysis.

The need to overcome these current limitations in the classic synthetic approach to strain engineering is highlighted by computational work on the benefits of dynamic strain modulation. Wittreich et al. showed by microkinetic simulations that ammonia synthesis on ruthenium nanoparticles could be significantly promoted by dynamic strain when applying uniaxial strain in the range of ±4% and using stimulation frequencies in the order of kHz (Figure 6c).^8^ While the proposed magnitude and time scale of dynamic strain modulation are beyond the current capabilities, further development in methods and catalytic materials may soon allow one to experimentally test such theoretical predictions.

### 5.1 Strain by Mechanical Forces

The generation and control of dynamic strain at material surfaces can be realized by several different means, depending on the targeted time/frequency ranges. In general, deformations in solids caused by external or internal mechanical forces are divided into regimes of elastic and plastic strain responses, depending on whether the solid returns to its original state after the forces have ceased (Figure 7b). In the following, we first consider dynamic elastic strains before discussing the potentials of controlled plastic deformations.

**Figure 7:**
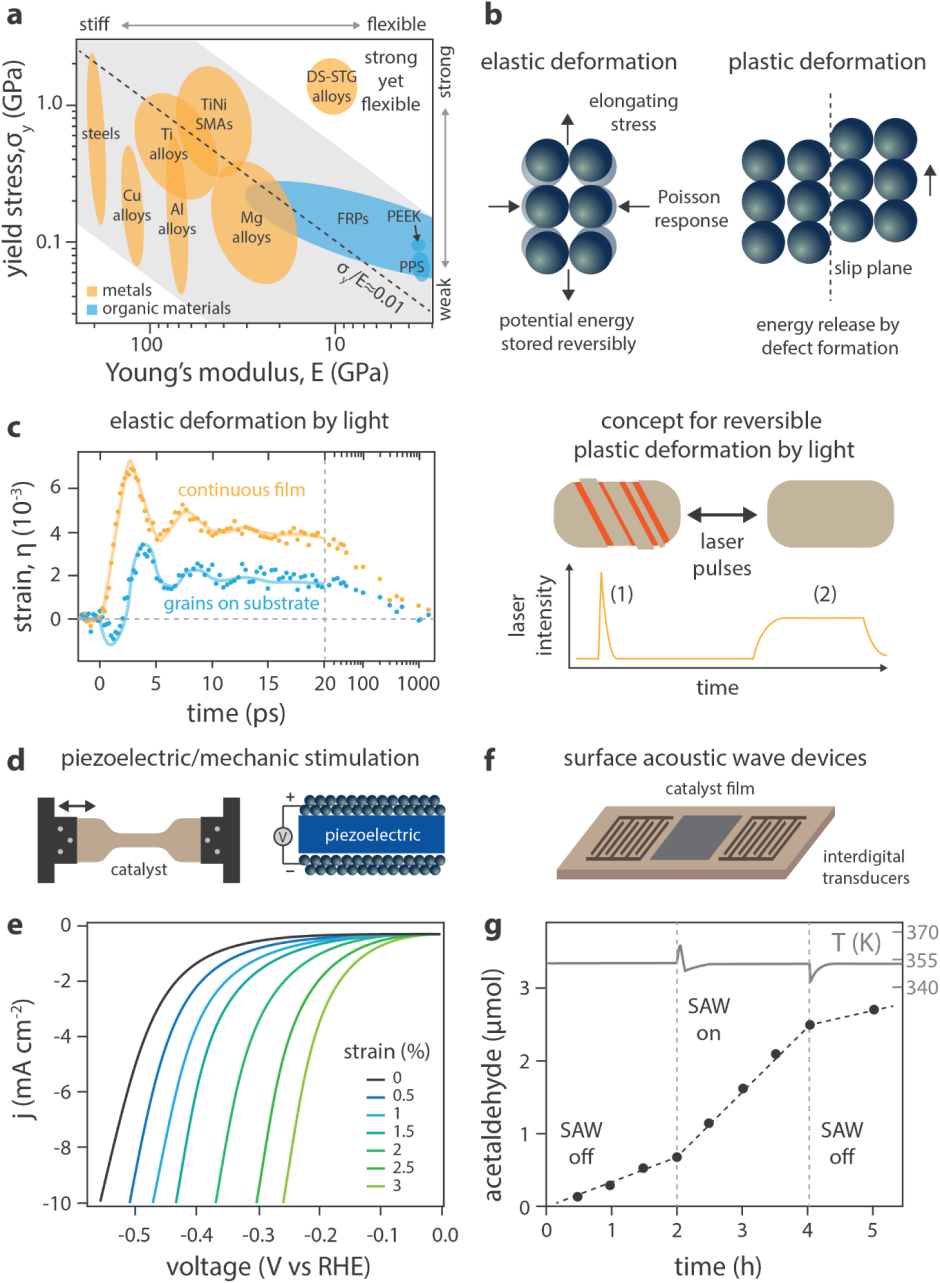
Materials and methods for programmable and dynamic strain in catalysis. (a) Yield stress vs elastic compliance for different materials, classified as strong and stiff, weak and flexible, and strong yet flexible. SMAs: shape memory alloys; DS-STG: dual-seed strain glass; FRP: fiber-reinforced polymers; PEEK: polyether ether ketone; PPS: polyphenylene sulfide. Adapted from Xu et al.^201^ (b) Schematic representation of elastic and plastic deformation in metals. (c) Using light to induce dynamic elastic and plastic strain in metal nanoparticles. Left: a 10 nm FePt film or FePt grains deposited on MgO are deformed by a laser, and the induced strain is measured by ultrafast X-ray diffraction in the ps regime. Adapted from Mattern et al.^202^ Right: concept to induce reversible plastic deformation in nanomaterials, using laser pulses of varying duration to cause defect formation and curing. (d,f) Methods to dynamically strain catalyst bodies using mechanical forces, piezoelectric materials, or acoustic waves. (e) Example of the effect of mechanically induced static strain on catalysis: linear sweep voltammetry (LSV) showing the Hydrogen Evolution Reaction (HER) current for rutile TiO_2_ films as a function of tensile stress. Adapted from Benson et al.^203^ (g) Example of the effect of dynamic strain introduced by surface acoustic waves (SAW) on catalysis: ethanol oxidation was accelerated over a 10 nm Pd film by 1 W, 19.5 MHz SAW. Adapted with permission from Inoue et al.^195^ Copyright 1989 American Chemical Society.

On relatively slow time scales (h/min/s), materials can be strained by applying external compressive or tensile mechanical forces, e.g., in dog-bone stretching devices (Figure 7d).^177,187^ Simultaneously, the catalytic activity can be monitored as a function of the direction and amplitude of the generated uniaxial, biaxial, or hydrostatic strain (Figure 7e).^177^ In this case, the strain is usually measured by mechanical or piezoelectric strain gauges. This approach is limited to relatively large length scales (down to millimeters) and to shaped catalysts, as the catalyst body must be clamped to a motor and physically stretched. Metallic foams or catalyst films supported on stretchable polymers are possible candidates for future studies in this direction (Figure 7a). Metallic glasses in particular are interesting, as they have a large rubber-like elastic strain domain (up to 8%) compared to metals.^187,201^ Moreover, the time scale for stretching can be shortened to ms using piezoelectric actuators^190,192^ to strain catalysts at frequencies in the region where resonant catalysis should be achieved.^19^

Alternative means to induce elastic strain on smaller length and time scales can involve functional materials exploiting piezoelectric, electro-, or magnetostrictive effects, such as piezo-/ferroelectrics or ferromagnets. The strain states of such materials and thus mechanically coupled catalysts can be dynamically and remotely controlled by time-dependent external electric and magnetic fields, respectively. A particular way to introduce large-amplitude acoustic dynamical strains at planar surfaces is given by so-called surface acoustic waves (SAW, Figure 7f). SAW amplitudes up to several Å can be reached by standard electronic means,^188,189^ and up to several nm using laser-excited SAWs.^191,193^ Well-defined high-frequency SAWs can propagate long distances, thus separating the generation area from the target area where the catalytic activity of a specific material is supposed to be promoted.^194^

SAW effects in catalysis were first reported in 1989 by Yasanobu Inoue and coworkers, resulting in a 2.6-fold enhancement of ethanol oxidation over Pd films (Figure 7g).^195^ Since then, many other examples of SAW-induced changes in catalytic performance have been reported for gas-phase reactions, showing up to one order of magnitude improvement in activity^196^ and changes in selectivity from 60 to 96% in ethanol decomposition to ethylene vs acetaldehyde.^197^ In some cases, a 2- or 3-order magnitude increase in activity was claimed, but notably starting from almost no initial activity.^198,199^

The mechanism for the enhancement of catalytic performance induced by SAWs remains unclear to this day. Several mechanisms were proposed, such as (i) electronic effects, (ii) dynamic coupling of vibrational modes with nonlinear components of SAWs, (iii) temperature effects, and (iv) structural changes in the catalyst film, such as intermixing. Recent studies on SAWs on Pt thin films revealed that the work function of the metal changed by about 0.5 meV, which suggests electronic effects on the binding energy of adsorbates can be ruled out.^194^ Similarly, photoluminescence experiments on a Cu-doped ZnS layer showed that the SAW produced an electric field of 9 × 10^3^ V cm^–1^ at 1 W power,^200^ which is several orders of magnitude lower than the electric fields, which can have an effect on adsorbate BE.^88^ Imperfections in the catalyst films were proposed to result in high (>10 Å) displacement among atoms, and even destruction of the films, due to phase shifts in traveling SAWs. SAW-induced intermixing was also observed in bimetallic metal films, suggesting that SAWs can induce structural reconstruction.^194^ Temperature effects are most probably at play, as changes in surface temperature up to 75 K were recorded for Rh thin films during SAW excitation.^194^

Regardless of the excitation mechanism, we note that the turnover efficiency estimated for a Pd thin film during SAW excitation was on the order of 10^–9^. This is consistent with the prediction of resonance catalysis theory that the efficiency of the excitation drops for high excitation frequencies,^32^ in the order of MHz for SAW devices. Ideally, one would want to fabricate devices that can resonate at different frequencies, in the range of kHz to MHz, to tune the stimulation to specific resonance catalysis applications. However, since the excitation frequency in SAW devices is inversely proportional to the thickness of the ferroelectric crystal, this seems unfeasible, as the surface area-to-volume ratio, a very important parameter for catalysis, would drop significantly. Nonetheless, fundamental studies on the mechanism of action of SAW devices can be valuable to understanding acoustic stimulation in catalysis.

### 5.2 Strain by Light Absorption

A very powerful and versatile approach to generate large-amplitude strains at very small length and time scales is by means of light absorption. For example, the absorption of intense laser pulses in solids usually causes the material to rapidly expand (Figure 7c, up to 0.6% strain).^204^ In ferroelectric or ferromagnetic materials, even laser-induced contraction is possible.^202,205,206^ As the laser-induced stress inside the excited material typically rises very fast (subpicosecond), acoustic strain waves are launched into adjacent materials and propagate to remote regions where they can act as an exclusive stimulus for catalysts, avoiding the need to disentangle thermal from elastic effects. Moreover, SAWs can be generated by laser-based transient grating and multiexcitation techniques, and their strain amplitudes can be boosted up to a few percent and even into the regime of plastic deformation (see below).^207^ The individual acoustic strain wave packets may involve acoustic frequencies up to hundreds of GHz, while the laser pulse excitation—and thus the strain modulation—can be periodically cycled at kHz and MHz repetition rates. Hence, these versatile laser-based approaches offer very broad frequency ranges of dynamic strain suitable for addressing a large variety of processes in resonant catalysis.

When a material experiences stress beyond a critical threshold, defects can form, leading to plastic deformation. Plastic deformation can directly alter active sites and, together with strain formation, modify the electronic structure of a catalyst. This is particularly relevant for nanomaterials, where high surface-to-volume ratios promote surface-dominated deformation pathways and plasticity-like atomic rearrangements that are inaccessible in bulk materials. In the case of photothermal reshaping of metal nanoparticles, such as gold nanorods, these structures in general deform toward their thermodynamically stable shape, accompanied by surface-facet reconfiguration that lowers the overall surface energy.^208,209^ Ideally, such structural changes would be reversible under dynamic stimulation, but under continuous or slowly varying heating, the evolution is typically dictated by thermodynamic driving forces.

Kinetic trapping provides a route to stabilize highly out-of-equilibrium atomic configurations. If heating and cooling occur on ultrafast time scales, as is the case for pulsed laser excitation, intermediate defect-rich and out-of-equilibrium structures can be formed and subsequently kinetically trapped. This enables the possibility of tuning atomic configurations via laser parameters such as pulse length, repetition rate, and fluence.^210^ Examples of such kinetically trapped states have been demonstrated: for instance, gold nanorods coated in mesoporous silica have been reshaped into highly strained nanocrystals with surface twinning defects while retaining their overall shape.^211^ The upper limit of the achievable frequency is determined by the time it takes atoms to diffuse into their new locations, which can be surprisingly fast. For gold nanoparticles, reshaping due to atomic diffusion was shown to be completed within a few tens of picoseconds.^211,212^

If reversible, such ultrafast strain modulation could be leveraged in dynamic catalysis. As schematically depicted on the right in Figure 7c, longer pulse excitations may allow stresses to be released in a controlled manner, potentially enabling reversible switching between different atomic configurations. Ongoing in situ TEM studies by some of us suggest that pulsed laser excitation can indeed induce a quasi-plastic regime in which reversible atomic diffusion and shape changes occur around a metastable, kinetically trapped state. This holds promise to extend the range of reversible dynamic stimulation beyond the elastic regime.

### 5.3 Summary and Outlook

In summary, we believe future efforts in strain-stimulated catalysis should be focused on achieving strain changes of at least 0.5%, in the frequency regime of kHz to MHz. In principle, this can be achieved by means of light stimulation in nanomaterials or mechanical stimulation of catalyst bodies. Ti-based alloys and metallic glasses are particularly interesting to achieve high strain values, as they have shown elastic deformations up to 8% under static conditions. However, it is largely unexplored what materials can withstand high strains under *dynamic* rather than static conditions. It might well be that other categories of material can withstand high strain under the short time scales in dynamic operation, and more materials research is needed to extend the current strain-resistant class of materials to dynamic operation conditions.

The main challenges that we foresee in this field are related to understanding how the mechanical and acoustic properties of the material influence the catalytic performance under dynamic stress. In current resonance catalysis models, it is assumed that a certain strain can be achieved at any given frequency, and the distribution of the strain in the material is assumed to be homogeneous. However, when stress is exerted on a body at a certain frequency, the resulting strain field distribution will depend on the dimensions, shape, and composition of the body. Depending on the frequency of the applied dynamic stress, the strain magnitude can be amplified at resonance frequencies, dictated by the eigenmodes of vibration of the body and therefore by, among other factors, the speed of sound in the body, its composition, and its dimensions and shape. Understanding and observing these effects will require a wide range of techniques, such as digital image correlation^213^ and full-field XRD^214^ to follow strain fields in shaped catalyst bodies, and time-resolved 4D STEM to study dynamically strained nanoparticles with atomic resolution at acquisition rates of μs.^215^ One can imagine that in order to promote catalysis effectively, the eigenfrequency of the catalyst body or nanoparticle must be properly “tuned” to the resonance frequency of the catalytic cycle.^32^ We believe this sets the scene for a new field of investigation at the intersection of catalysis, mechanical engineering, and acoustics.

## 6 Dynamic Catalysis Triggered by Heat

In both industrial and research practices, the dominant approach has been to optimize the chemical kinetics and thermodynamic equilibrium for continuously heated reactors. Within this paradigm, temperature is treated as a static or semistatic variable due to constraints imposed by system and reactor designs,^216^ rather than by choice. Although the virtues of dynamic catalyst heating have been postulated since the late 1960s,^217^ bulk-scale reactors that are heated by steam or via fuel combustion suffer from substantial thermal inertia, with typical heating and cooling cycles on the order of hours. Recent advancements in dynamic and pulsed heating techniques, in which the catalyst temperature is temporally modulated on a subsecond time scale, have allowed exploration of rapid heat-triggered dynamic catalysis. Before discussing the mechanisms of how dynamic heating can affect the reaction rate, energy efficiency, reaction selectivity, and catalyst stability, we first discuss how the choice of heat source and the sample’s dimensions determine the accessible heating and cooling rates.

### 6.1 Heating Methods and Dimensional Effects

Catalysts can be directly or indirectly heated through a wide variety of mechanisms, such as fuel combustion, electric current (Joule or resistive heating),^218^ mechanical work, electric fields (e.g., plasma and dielectric heating), magnetic fields (induction),^219^ and through the decay of optically excited carriers or vibrations.^158^ Thus far, dynamic heating of catalysts has been explored using Joule, photothermal, and microwave methods, each with their own possible realms of heating and cooling rates and effective volume (Figure 8).

**Figure 8:**
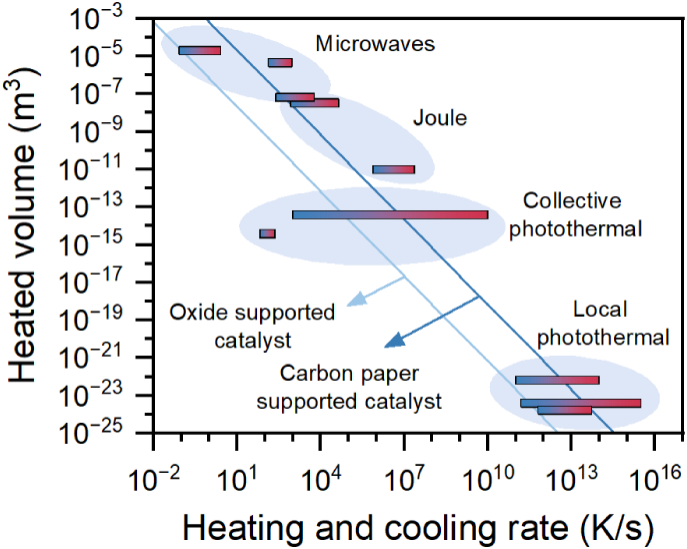
Pulsed heating for dynamic catalysis. Literature examples of heating and cooling rates (red and blue ends of the gradients, respectively) obtained with different pulsed heating technologies: microwaves,^220–222^ Joule heating,^223–227^ collective photothermal effects,^228,229^ and localized photothermal effects.^229–231^ The rates are plotted according to the size of the heated volume, highlighting the inverse scaling relation between sample dimensions and maximum rates of temperature change. The two blue lines represent cooling for internally diffusion-limited heat transfer for a spherical volume of an oxide-supported (light blue) and carbon-paper-supported catalyst (dark blue), based on the relationship $\tau =\frac{L^{2}}{\alpha }$ where τ is the time constant for cooldown, *L* is defined as the radius of the heated volume, and α is the thermal diffusivity of the catalyst (estimated as 10^–7^ m^2^/s for a loosely packed metal oxide catalyst and 10^–5^ m^2^/s for a carbon-paper-supported catalyst). The cooling rate is then given by $\frac{\Delta T}{\tau }$ , with Δ*T* arbitrarily set to 300 K.

Joule heating, also known as resistive heating, is the process where electric current that passes through an electrical resistor converts into heat.^218^ Pulsed Joule heating has so far been shown to drive dynamic catalysis by heating and cooling cycles in a micro- to millisecond time range.^223–226,232,233^ The observed heating rates can reach up to 1 × 10^7^ K/s (Figure 8) but are strongly dependent on the intrinsic properties of the heating material. For the same electrical current, heater dimensions, and electrical pulse width, the heating rate is often inversely correlated with the material density, heat capacity, and heat conductivity, where faster heating rates are typically observed with heater materials such as carbon, some silicon carbides, and stainless steel.^226,234^ For achieving the fastest electrical pulses, pulse generators based on capacitor-discharge circuits are sometimes more effective.^234,235^ Note that a trade-off may be made between the ramping rate, high-temperature stability, and mechanical strength of heater materials.

Photothermal heating uses light illumination in combination with materials that efficiently convert light to thermal energy through electronic excitation and decay of excited states by coupling with phonon or vibrational modes. The best materials of choice feature strong light-matter coupling, high absorption cross-sections, low scatter cross-sections, strong electron–phonon coupling, and a suppression of radiative decay pathways, such as in the case of plasmonic nanoparticles, metal nitrides and carbides, indirect semiconductors, and carbon materials.^158^ The photothermal material may be the catalyst as well, or it can be in close contact with the catalyst, such as in antenna-reactor complexes.^236^ Crucially, using pulsed light can give access to the highest possible heating rates, up to 10^14^ K/s under fs-pulsed illumination,^212,230,237,238^ and allows for elaborate control and programming. Cooling rates depend strongly on the surroundings of the catalyst but can reach up to 10^11^ K/s for single nanocatalysts.^230,231^

Thus, pulsed illumination produces short, intense, and localized heat bursts interspersed with cooling intervals that have fast heating and cooling rates, enabling high control over both spatial and temporal localization of heat. In this way, different processes in catalysis reactions can be favored in heating/cooling periods, which brings possibilities to manipulate the processes through adjusting the parameters of pulsed light. On the other hand, the largest challenge in using pulsed light effectively at a larger scale is to illuminate the photothermal catalyst controllably in larger volumes. Notably, solutions to illuminate photocatalysts in large-scale reactors are already in development, with promising concepts including optical fibers, light guides, flow reactors, and reticulated solids.^173^ Such reactors may also require less heat management and waste-heat recovery strategies.

Microwaves between 0.3–300 GHz (λ = 1 mm–1 m) generate heat in heterogeneous catalysts primarily through three mechanisms: (1) dielectric heating, where oscillating electric fields cause dipolar polarization and ionic conduction; (2) magnetic heating, involving interactions between the microwave’s magnetic field and magnetic materials; and (3) conduction loss heating, where induced currents in conductive materials lead to Joule heating.^239,240^ Pulsed microwave heating has been investigated since the 1990s, which can lead to a dynamic mode of operation.^220^ Depending on the heated volume, heating and cooling rates can be achieved up to 1 × 10^3^ and 1 × 10^2^ K/s, respectively. Higher heating rates are within reach by using microwave lenses and local resonance effects, analogous to optical and nanophotonic technologies.^241^

Pulsed microwave heating is currently limited to two aspects. First, even for CW microwave excitation, it is already challenging to differentiate between various activation mechanisms. The addition of pulsed modulation creates an extra layer of complexity that calls for the careful interpretation of data and meticulous control experiments. Second, further research is needed to develop scaled-up reactors and pulsed microwave generators that remain cost- and energy-efficient.^242^ Nonetheless, pulsed microwaves are a highly attractive form of reactor heating and can be directly adapted for use in dynamic catalytic systems.

### 6.2 Scaling Relationships of Thermal Transport

For each of these pulsed heating methods, the ideal scenario is one in which only the catalyst is selectively heated and its temperature is modulated faster than the kinetics of the targeted process of chemical transformation. This not only ensures higher energy efficiency but can also prevent undesired reactions in the surrounding medium. In general, pulsed heating methods can reach much higher temperatures than their continuous counterparts due to the transient nature of the energy input.

A key constraint in pulsed thermal control is the size-dependent nature of the heat transport for both heating and cooling. The accessible heating and cooling rates are governed by three primary factors: the volume of the heated region, the peak power density available, and the thermal diffusivity of the catalyst material ( $\alpha =\frac{\kappa }{\rho C_{p}}$ , where κ is the thermal conductivity (W/m·K), ρ is the density (kg/m^3^), and *C*_*p*_ is the heat capacity (J/kg·K)).

Fundamentally, both the heating and cooling rates are inherently dependent on the catalyst sample size (Figure 8). The heating rate ( $\frac{\Delta T}{\tau }$ in K/s) before losses occur scales with the available power (*P*, J/s) and inversely with the thermal mass (*mC*_p_) through $\frac{\Delta T}{\tau }=\frac{P}{mC_{p}}$ . This relationship dictates that it is physically challenging to heat the catalyst in a nearly instantaneous fashion on a large scale. For example, ultrafast heating time scales in the picosecond range are typically achievable only at the smallest length scales,^229^ such as micrometers or smaller, while longer time scales, on the order of milliseconds, are associated with larger, submeter scales (Figure 8).^225,232,242^

While previous literature has often emphasized the heating phase, the cooling step, including both rate and duration, also plays a critical role in determining the overall effectiveness of pulsed heating approaches, though this aspect remains relatively unexplored. In the diffusion-controlled regime, the cooling rate ( $\frac{\Delta T}{\tau }$ in K/s) scales with the thermal diffusivity and scales inversely with the square of the characteristic length of the catalyst volume (*L*) due to the relationship $\tau =\frac{L^{2}}{\alpha }$ . Thus, the fastest cooling rates are achieved at the smallest length scales. This relationship indeed holds well for a wide range of pulse-heated catalytic methods reported to date (Figure 8). This illustrates that the cooling rate is the primary physical constraint that imposes a fundamental limit on how rapidly catalyst temperature can be switched down, especially at larger scales. Therefore, successful scale-up of pulse-heated catalytic systems requires careful co-optimization of catalyst geometry, reactor design, throughput requirements, and available heating power. To enable rapid cooling rates, materials with higher thermal conductivity and structures enabling faster heat diffusion should be considered, such as carbon derivatives and 2D materials.^243^ These considerations become increasingly important, as such technologies could approach higher technology readiness levels.

### 6.3 Mechanisms in Pulsed Heating of Catalysts

There is theoretical and experimental evidence that pulsed heating of catalysts can lead to (1) higher reaction rates and energy efficiency, (2) steering of reaction selectivity, (3) removal of catalyst poisons, (4) avoidance of side or sequential reactions, and (5) improvement of catalyst stability. Using literature examples, we will discuss and illustrate the underlying mechanisms.

It is important to note that, contrary to other dynamic catalysis approaches, pulsed heating alone does not alter the binding energies between adsorbates and catalyst but transiently modulates the availability of thermal energy.^38^ This modulation has the power to partially decouple and independently optimize key physical transport mechanisms, such as mass flow and adsorption–desorption equilibria, from chemical reaction steps occurring at active sites, which are dictated by activation energies and chemical equilibrium. By delivering transient energy inputs over short time scales aligned with (i) intermediate reaction steps, (ii) heat generation and transfer processes, or (iii) surface adsorption and desorption dynamics, pulsed heating selectively activates or suppresses specific reaction pathways. This enables dynamic control of the reaction progress, adjustment of selectivity, and tuning of reactivity (Figure 9).

**Figure 9:**
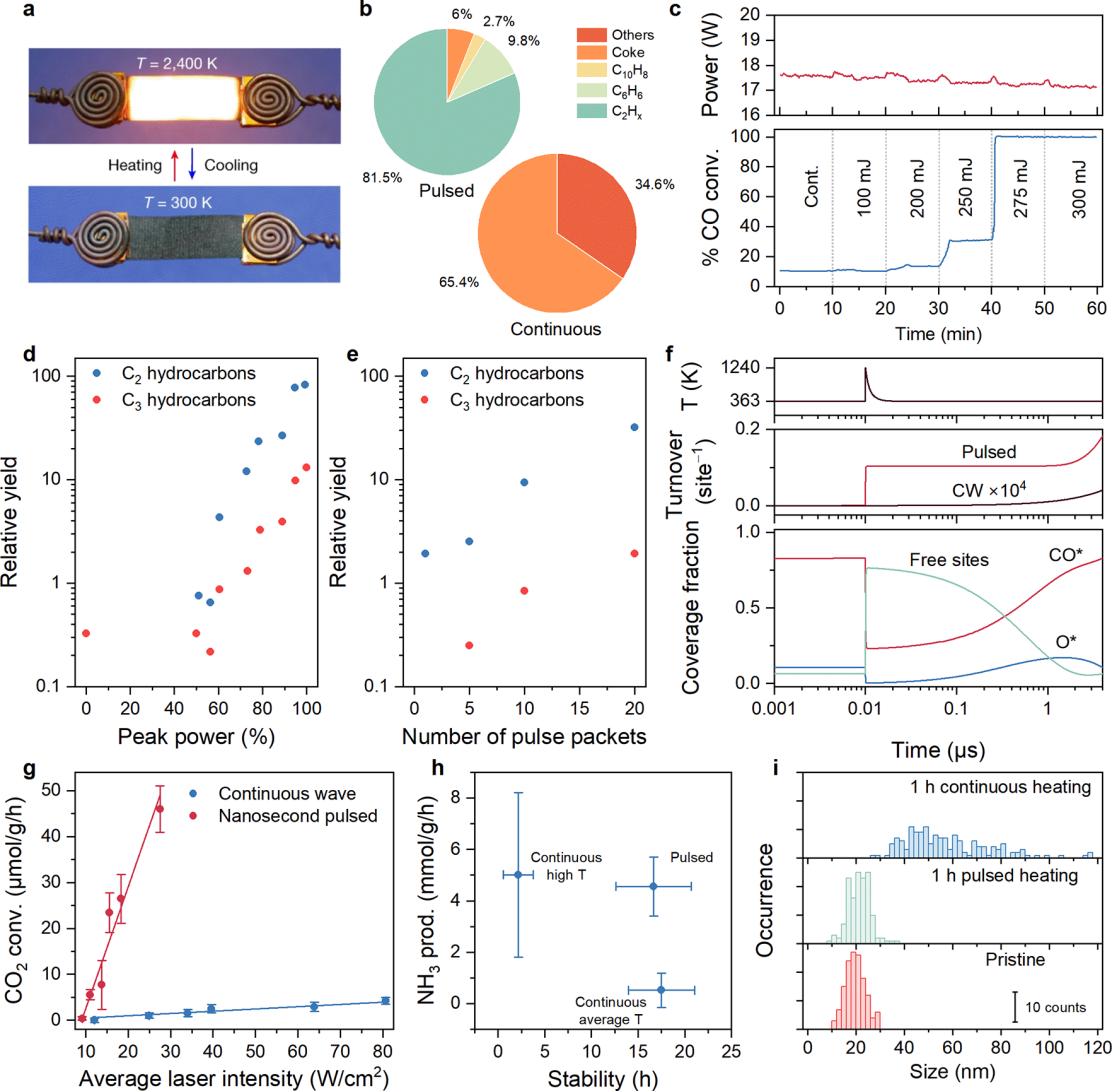
Pulsed catalyst heating for controlling turnover, selectivity, and stability. (a) Example of a Joule heater, with carbon paper clamped between two electrodes.^225^ (b) Comparison of product selectivity for pulsed and continuously heated methane pyrolysis. Data extracted from Dong et al.^225^ (c) Conversion of CO oxidation on a Pt-decorated Joule heater under continuous (0–10 min) and pulsed heating (10–60 min). The total power input is shown in the upper panel. Data extracted from Zhu et al.^224^ (d,e) Relative yields of C_2_ and C_3_ hydrocarbons during pulsed microwave heating of Ni-catalyzed methane pyrolysis, as a function of pulse peak power (d) and the number of pulse packets (e). For panel (d), 20 × 240 ms microwave pulse packets were applied with a delay of 5.76 s between packets. For panel (e), a constant total irradiation time of 5 s (constant total energy input) was divided into various numbers of pulse packets. Data extracted from Wan et al.^220^ (f) Microkinetic modeling of CO oxidation under ultrafast photothermal excitation (at 250 kHz and 200 W/cm^2^): the bottom panel shows time-dependent adsorbate coverages during a single pulse period, the middle panel shows the integrated turnover, and the top panel shows the temperature evolution. Adapted with permission from Baldi and Askes,^229^ Copyright American Chemical Society 2023. (g) Catalytic CO_2_ conversion rate on Au-ZnO as a function of average laser intensity under continuous wave and 5.5 ns pulsed illumination at λ = 532 nm. Data extracted from Wang et al.^245^ (h,i) Catalytic activity and stability during pulsed NH_3_ synthesis on a carbon-Ru catalyst at continuous heating (high *T* = 1400 K, average *T* = 900 K) and pulsed heating (switching from 700 to 1400 K (averaging 900 K), 0.11 s on, 0.99 s off). Panel (i) shows the size distribution of the Ru catalyst, where 1 h of pulsed heating minimally affects the distribution compared to continuous heating. Data extracted from Dong et al.^225^

First of all, pulsed heating can exploit the time scale differences between adsorption–desorption dynamics (μs–s) and surface reactions and catalytic cycles (ps−μs), see Figure 2e.^170^ As a prominent example, consider the model reaction of CO oxidation on Pt, which is normally severely limited due to the strong binding of CO (i.e., surface poisoning) and consequential low oxygen coverage. Upon subjecting the catalyst to an intense heat pulse, the surface becomes depleted, and new reactants can adsorb at rates that correlate with their impingement frequency and sticking factor.^216^ Crucially, these adsorption rates determine the initial distribution of adsorbed species (ns−μs regime), whereas the equilibrium coverages are reached at longer time scales (μs–s, depending on temperature and binding energies), as dictated by the difference in binding energies. In the case of CO oxidation, this means that oxygen coverages are transiently much higher until equilibrium is reached, leading to a strongly enhanced reaction rate. Indeed, it has been experimentally demonstrated that pulsed Joule heating of a Pt film (25 μs pulse, 2.3 × 10^7^ K/s, 50 ms duty cycle, 230 K amplitude) led to a 40-fold higher CO oxidation rate compared to continuous heating with the same power input (Figure 9c).^223,224^ This was further corroborated by a simulation study of pulsed photothermal catalysis using plasmonic nanoparticles (Figure 9f).^229^ This example of CO oxidation highlights that poisons can be removed by periodic heat pulsing, which could be an effective strategy for the regeneration of poison-deactivated catalysts.

A further advantage is that between heat pulses, the catalyst experiences a much lower temperature, at which the catalyst can reach high reactant loading. Since the reaction rate is the product of the reaction constant and surface coverages, a rapid heat pulse can drive catalysis at a higher turnover rate than under steady-state conditions, for which reaction rate and surface coverages are mutually exclusive.^216^ By matching pulse parameters with the underlying dynamics of adsorption, a resonance can be reached, where reaction rates and energy efficiency are optimized.

The transient out-of-equilibrium distributions of surface species are ordinarily out of reach under any steady-state temperature.^224,229^ Pulsed heating can therefore be regarded as a new tool to directly control surface coverage of reactants and intermediates. On the one hand, this can lead to increases in reaction turnover and energy efficiency, as in the case of CO oxidation. On the other hand, since the distribution of surface coverages is intimately linked to the selectivity, there are additional opportunities to control the selectivity in more complex reaction networks. These effects have thus far been explored in theory,^229,244^ which motivates experimental validation.

A further benefit of pulsed heating is that reactions can be rapidly started and equally rapidly quenched, which allows for the prevention of undesired sequential reactions. This, in turn, can lead to major differences in the reaction selectivity. For example, it was shown that pulsed Joule heating of methane pyrolysis on a porous carbon heater (0.02 s pulse, 1.1 s duty cycle, 1400 K amplitude) prevented the formation of coke and low-value products (Figure 9a,b).^225^ By optimizing the pulse amplitude and duration, the selectivity could be tuned to almost exclusively yield high-value products (e.g., C_2_ species and benzene). Similar observations were made approximately 30 years ago by powering methane pyrolysis using pulsed microwave heating (Figure 9d,e).^220^ With the same average input power, different conversion rates and product distributions were obtained by varying the pulse amplitude and repetition rate. Note that this valuable strategy relies on quenching the reaction early on so that sequential reactions that occur at longer time scales are prevented and the most valuable products can be collected.

Furthermore, forms of specific and localized heating (e.g., pulsed light and microwaves) can result in higher reaction rates due to a much smaller affected volume. For the same power, this leads to much higher ramp rates or peak temperatures. The exponential relationship between reaction rate and temperature (the Arrhenius equation) leads to greatly enhanced reaction rates during the pulses, which can offset the idle time between pulses. For example, it was found that pulsed illumination (532 nm, 5.5 ns pulses) of a photothermal CO_2_ hydrogenation catalyst (Au/ZnO) at the same laser intensity and wavelength resulted in a 50 times higher conversion rate than CW illumination (Figure 9g).^245^ Similarly, it was found that pulsed microwave heating required 4 times lower power input compared to continuous heating for the same product generation in the synthesis of 5-hydroxymethylfurfural.^221^

Pulsed heating often introduces spatial temperature gradients and, in some cases, distinct temporal temperature variations across different spatial locations within the reactor. These effects can result in reaction inhomogeneity, which may or may not be desirable depending on the specific application. Many of these additional effects remain underexplored and represent promising areas for future research. Already under steady-state heating, large thermal gradients (up to 90 K) can exist between adjacent regions at the micro- and nanoscale,^246,247^ which are recognized to have implications for catalytic performance. Obviously, the introduction of an external transient stimulus leads to additional complexity. For example, adjacent regions with higher and lower local temperatures may exhibit differences in activity and selectivity, which can be used to partition different reaction pathways across a surface for tandem catalysis. Transient heat gradients may influence mass transport by coupling with convective flows or diffusion and may facilitate the diffusion of trapped reactants and intermediates toward active sites.

From a reaction engineering perspective, pulsed heating offers significant benefits for improving energy efficiency, particularly in exothermic catalytic reactions.^248^ In such cases, the heat generated by the reaction itself can partially or even fully sustain the activation energy requirements (also known as catalytic ignition),^249^ thereby saving external energy inputs. Additionally, in flow-type thermochemical reactors, repetitive cooling during exothermic catalytic reactions can induce a favorable equilibrium shift toward higher reactant conversion, as predicted by the van’t Hoff equation. On the other hand, endothermic reactions can in turn benefit from pulsed operations, as the reaction consumes thermal energy, which may lead to faster cooling rates. In this context, both types of reactions require a different strategy for engineering the thermal dissipation at the catalyst sites in order to tune the length of the heating phase.

Additionally, pulsed heating can contribute to the stability of heterogeneous catalysts, which usually deactivate through various mechanisms such as sintering, random migration, and phase segregation.^250^ Thanks to the very short interval of heating and the much slower time scale of such deactivation mechanisms, the catalyst becomes markedly more stable over time.^225,226^ For example, Ru nanoparticles on carbon support remained distinctly more stable for pulsed Joule heating (0.11 s pulses, 1.1 s duty cycle, ∼700 K amplitude) compared to steady-state heating (Figure 9h,i).^225^ Likewise, pulsed Joule heating of a PtNi/SiO_2_ catalyst (50 ms pulses, 1 s duty cycle, 700 K amplitude) for dry reforming of methane suppressed coke formation, sintering, and phase segregation.^226^ Further, heat pulses can potentially regenerate spent catalysts and lead to the in situ formation of active species. For example, pulsed heating was demonstrated to result in disintegration of sintered clusters and larger, inactive nanoparticles.^251,252^

Finally, transient heating effects can also lead to changes in the catalyst’s potential energy landscape by inducing strain, defect deformation, changes in local atomic arrangements (e.g., adatom diffusion), (re)­construction of active sites, formation of facets and domains, extreme electronic temperature, pyro-catalytic effects,^253^ or charge evolution (e.g., low-temperature plasma). For example, photoexcited carriers and transient radicals and ions in combination with heat can lead to new ways of catalytic control.^253–255^ These dynamic changes can give rise to unconventional and potentially advantageous or even synergistic catalytic properties that cannot be accessed under steady-state conditions.

### 6.4 Optimal Heat-Pulse Parameters

An open question concerns the optimal duration of the heating pulses. Importantly, the ideal pulse duration is likely to be highly system-dependent and determined by the reaction kinetics, which determine the lower limit for the required heating period. Reactions involving inherently slow kinetics, such as those with high-entropy transition states or significant structural rearrangements, may not proceed effectively under very short heating intervals (fs-ps regime). For example, in the case of pulsed-heated enzymes, it was found that fs-pulsed illumination (with the fastest heating rate and maximum peak temperature) was outcompeted by ns-pulsed illumination^256^ because the structural rearrangements that govern enzymatic cycles require at least ns intervals. Similar relationships may hold for heterogeneous catalysts, which calls for systematic study of the influence of pulse duration on dynamic catalytic performance.

A similar relationship holds for the optimal pulse repetition rate. The ideal pulse frequency will be determined by the kinetics of local mass-transport phenomena, in particular, the diffusion, adsorption, and desorption of reactants, intermediates, and products. In the limit of a too-high pulse frequency, diffusion and adsorption of reactants cannot occur, leading to a hot but empty catalyst. In different scenarios, products that are still on the catalyst would experience another pulse too soon, leading to a sequential and undesired reaction (e.g., coke formation or product decomposition). In the case where the pulse frequency is too low, the adsorption–desorption equilibrium could converge to that of the steady state, which would avoid some of the benefits of pulsed heating. These considerations should motivate researchers to optimize the repetition rate systematically by designing experiments with frequency-tunable heat sources. In addition, introducing a complex, programmed temperature profile with variable and deconvoluted time scales could unlock new catalytic pathways beyond what can be achieved via simple pulse patterns.

### 6.5 Challenges and Opportunities for Heat-Driven Dynamic Catalysis

The diverse methods and mechanisms of pulsed heating provide a valuable platform for driving catalytic reactions beyond steady-state limitations as well as gaining fundamental insights into the ultrafast processes governing these reactions. From a heterogeneous catalysis perspective, pulsed heating enables precise control over intermediate reaction steps and/or superficial adsorption–desorption processes as well as controlling structural processes that affect stability. The most promising results so far have been acquired using pulsed Joule heating, which we also identified to offer relatively longer time scales in the range of milliseconds. These results have already provided valuable insights into the possibilities of this research field and provided motivation to continue exploring faster time scales and various stimuli. Nonetheless, we speculate that more striking benefits could be achieved in photothermal heterogeneous catalysis when tunable and transient heating on ps–ns time scales can be implemented. However, the opportunities in this area are accompanied by numerous challenges that still need to be overcome, which we summarize below.

Experimental studies have begun to explore pulsed thermal catalysis, yet the field is far from achieving the theoretically predicted orders-of-magnitude catalytic improvements. A significant barrier lies in the vast parameter space, encompassing variables such as pulse duration, frequency, intensity, and wavelength, on top of typical parameters of catalytic setups. The parameter landscape becomes even infinitely more complex with variations in the pulse-to-pulse intensity, duty cycle, duration, and waveform. Considering this overwhelming complexity, it is clear that advances in machine learning and artificial intelligence could be powerful methods to navigate and optimize this space and significantly accelerate progress, which has already been demonstrated for millisecond pulsed Joule heating.^225^

Another factor of complication is that a variety of mechanisms can play a role, as highlighted in Section 6. This motivates the design of simple experiments with model reactions that can clearly distinguish between competing effects, as well as a cautious attitude in interpreting results, before moving on toward more complicated systems. New methods of measuring dynamic catalytic processes with sufficient time resolution should be used (see Section 7) to provide convincing evidence of novel claims.

An overarching challenge in the field lies in the inherent spatiotemporal limitations of introducing and dissipating heat in a controlled way. Increasing the system size and stimuli frequency is strongly limited by the cooling rate of the catalytic ensemble to ensure the desired effect of each pulse. These trade-offs are visualized in Figure 8, which maps current technologies on a spatiotemporal scale, revealing critical gaps where no currently explored method achieves both high temporal and spatial control. For instance, Joule heating can generate thermal pulses of 2000 K in 0.02 s on a cm^2^ reactor dimension but is constrained by a slow heat dissipation. On the other hand, photothermal heating on plasmonic nanoparticles can achieve much shorter heat pulses in the ps regime but is limited by collective heating effects that limit the temperature contrast between light-on and light-off states.^229,257^ It was shown that such collective heat, lingering in the material between pulses, can counter the beneficial effects of heat pulsing by accelerating adsorption and desorption toward equilibrium.^229^

Successful implementation of pulsed heating technologies for heterogeneous catalytic reactions therefore requires precise control over the cooling dynamics. Achieving this requires an integrated approach that combines advanced material design and architecture (e.g., microporous catalysts or substrates), innovative reactor engineering (e.g., active cooling),^248^ and meticulous process optimization (e.g., selective electromagnetic and induction heating). Lab-scale experiments should go hand-in-hand with multiscale modeling of heat dissipation at the nanomicroscale catalyst level and at the reactor level, which can be readily adapted from literature on photothermal catalysis and temperature jump experiments.^258,259^ Heat dissipation strategies developed in the chip manufacturing industry, where high-end processors dissipate vast power consumption in minuscule volumes (>1000 W/cm^3^), could provide valuable lessons for rapid progress on these challenges.

In summary, although pulsed heating in catalysis faces considerable challenges, the field is evolving rapidly and holds immense promise. We expect significant progress by addressing the challenges of introducing and removing heat from the catalyst with fast time resolution, navigating the complex landscape of pulse parameters, and distinguishing between competing dynamic mechanisms. Simultaneously, these challenges underline the need for a multidisciplinary approach, where existing knowledge from fields such as physics, chemical engineering, mechanical engineering, and materials science should be effectively combined to push the field forward.

## 7 Methods for Studying the Mechanisms of Dynamic Catalysis

The prerogative of stimulated dynamic and resonant catalysis is to use an external stimulus to change the catalytic system over time, for example, by heating, photoexcitation of charge carriers, straining, or charging of catalytic surfaces and interfaces, with the aim of boosting catalytic performance. Theoretical models predict that this can be achieved by changing the surface coverage and/or the binding energy of reaction intermediates, working outside of steady-state conditions. Experimentally, evidence of the enhancement of catalysis by dynamic stimulation is growing. However, a theoretically predicted improvement in catalytic performance of orders of magnitude was not yet realized. The reason for this gap between the experiment and theory is unclear and requires further study.

While different levels of theory (e.g., DFT, MD) can be used to simulate the effect of a certain stimulation on catalytic systems, a complexity gap exists between the theories of dynamic catalysis and their real-world application: theory makes use of model systems, i.e., well-known reactions at a single or few active sites. In reality, a catalyst consists of multiple active sites and interfaces, which can react differently to stimulation and changes in conditions and operate according to complex reaction networks involving different pathways and multiple reaction intermediates.

Experimentally, realizing dynamic and resonant catalysis therefore introduces a number of challenges related to the interaction of a certain stimulus with a catalytic material (or device) of choice under a set of working and stimulation conditions. Such challenges call, on one hand, for more extensive and precise models and, on the other hand, for the development of characterization techniques to better understand the mechanism by which a catalyst is stimulated and experimentally validate theoretical models. Moreover, correlating stimulation-induced changes in catalyst structure, composition, and/or surface chemistry to catalytic performance would ideally provide guidelines to explore the vast parameter space of catalyst stimulation (nature of stimulus/stimuli, frequency, intensity, duty cycle, phase delay, etc.), especially in combination with high-throughput methods and machine learning algorithms.

In this section, we will first propose the concept of stimulus design (next to traditional catalyst design), which describes the importance of optimizing external stimuli to enhance the catalytic performance. Then, we introduce *stimulando* characterization as an approach to study catalysts under both stimulation and reaction conditions, linking structural changes to performance. We finally discuss the technical challenges and the potential of such an approach in yielding new insights into dynamic catalysis.

### 7.1 From Catalyst Design to Stimulus Design

For decades, the choice and development of improved catalysts for a specific chemical reaction followed the paradigm of catalyst design, that is, a process based largely on trial-and-error and partially guided by a fundamental understanding of the chemistry and mechanism as a function of catalyst composition and structure. While the choice of the catalytic material (or device) remains an important part of the picture in the case of stimulated dynamic and resonant catalysis, the choice of effective stimulation becomes just as important in achieving an improved catalytic performance. We can therefore introduce the concept of stimulus design, that is, a process of optimization of catalyst stimulation, based on trial-and-error and partially guided by knowledge of the effect of stimulation on catalysis. While optimizing the stimulation for a certain catalyst-reaction pair can be achieved by brute force, exploring parameters using high-throughput and statistical methods, we believe that a fundamental understanding of stimulus-catalyst interaction based on characterization methods can help (i) translate the knowledge for a given system to others and aid in the selection of different, and more promising, stimulus-catalyst combinations and (ii) provide feedback and input to theoretical models used to predict the effect of catalyst stimulation.

In this context, we wish to know:

- Which reaction intermediates and/or catalyst components respond to a stimulus, and which do not do so appreciably?
- How are reaction intermediates affected in terms of the change in binding energy and coverage (possibly in time and space)?
- How fast does the stimulation affect the catalyst and different reaction intermediates?
- How do the changes in surface chemistry affect the catalytic activity in terms of average and time-resolved TOF?
- How (ir)­reversible are these changes?

In other words, we are concerned with how, how fast, and how much we can alter the energy landscape of a given reaction network over a certain catalyst and to what extent the changes imparted are affecting catalytic performance under the given conditions. Depending on the stimulus of choice, we may be interested in different chemicophysical aspects of the interaction with the catalyst: for light, charge carrier dynamics; for electric fields and charges, charge distribution and electronic properties of the catalyst surface; for stress, strain distribution and magnitude; for heat, local temperature and gradients. Regardless of the chosen stimulation method, decoupling effects between different stimulation pathways (e.g., when using light, you potentially have heat, strain, and charge effects) could help to guide better catalyst stimulation. This is a spectacular challenge, for which the operando characterization methods developed over the last decades shall be used as a logical starting point.

### 7.2 State-of-the-Art in In Situ and Operando Characterization of Catalytic Systems

Understanding how catalytic materials function is challenging because of numerous entangled effects that govern their performance. Reaction conditions, active sites, intermediates, spectating species, deactivation processes, diffusion limitations, and other phenomena interact dynamically, making working catalysts exceptionally complex materials. As Francis Crick famously said: “*If you want to understand function, study structure*”. Because the structure of catalysts is highly sensitive to the reaction conditions, a transformative step for catalysis research has been the development of characterization techniques in situ and operando—defined as the simultaneous characterization of a catalyst under working conditions, alongside performance measurement.^260–264^ Such characterization approaches have led to remarkable advances in the molecular understanding of catalysts over the past four decades.

In situ and operando characterization of catalysts has predominantly relied on photons. For example, vibrational spectroscopy techniques, such as infrared and Raman spectroscopy, are commonly used to study the structure and coverage of reaction intermediates, while X-ray-based methods, including X-ray absorption and scattering, are indispensable for resolving the structure of active catalytic phases. In recent years, remarkable developments in electron microscopy and (near-)­ambient pressure X-ray photoelectron spectroscopy have made these techniques more common and valuable tools to analyze working catalysts.^265^ Furthermore, more exotic techniques—ranging from operando solid-state NMR spectroscopy^266^ to single-molecule fluorescence microscopy^267^—have provided important insights into various catalytic phenomena.

Despite its notable success, conventional in situ/operando methodology often focuses on static, one-dimensional reactivity descriptors. This approach overlooks the inherently dynamic interactions between reaction intermediates and active sites, which are central to catalytic function.^268^ Addressing this limitation requires the adoption of dynamic operando characterization methods, such as modulation-excitation (ME)—often coupled with phase-sensitive detection (PSD)—and Steady-State Isotopic Transient Kinetic Analysis (SSITKA) coupled with spectroscopy methods. Such approaches can distinguish active species from spectators and help to capture the temporal evolution of catalytic systems, offering a more complete understanding of their complex behavior. Transient characterization methods can be readily used or adapted to study dynamic catalysis. For example, Diffuse Reflection Infrared Fourier Transform Spectroscopy (DRIFTS) was recently used to understand the effect of pulsed heating on Ni-based catalysts for the activation of CO_2_ in the presence of H_2_, where a shift in selectivity from methane to CO was observed upon pulsed heating.^227^ Moreover, pump–probe spectroscopy methods can be used to probe ultrafast phenomena (down to fs scale)^269^ and can be potentially adapted to study the effect of dynamic stimulation on catalysts, using the stimulation as a pump and triggering the spectroscopic probe at different phase delays. The potential and challenges of such an approach are discussed below.

### 7.3 Introducing “Stimulando” Characterization

The methods of operando characterization described above can, in principle, be readily applied to the study of stimulated catalysis (Figure 10a,b). Nonetheless, there is an additional layer of complexity to take into account when one wants to understand the mechanism and effects of a certain stimulation on catalysis. Namely, one wants to

- Deliver the stimulus to the catalyst while acquiring spectroscopic or characterization data under reaction conditions.
- Sample the part of the catalyst material or device affected by the stimulus to observe changes in the catalyst and catalytic mechanism during stimulation.
- Monitor the performance of the stimulated catalyst to determine the effectiveness of a certain stimulation and correlate this with spectroscopic signatures.

**Figure 10:**
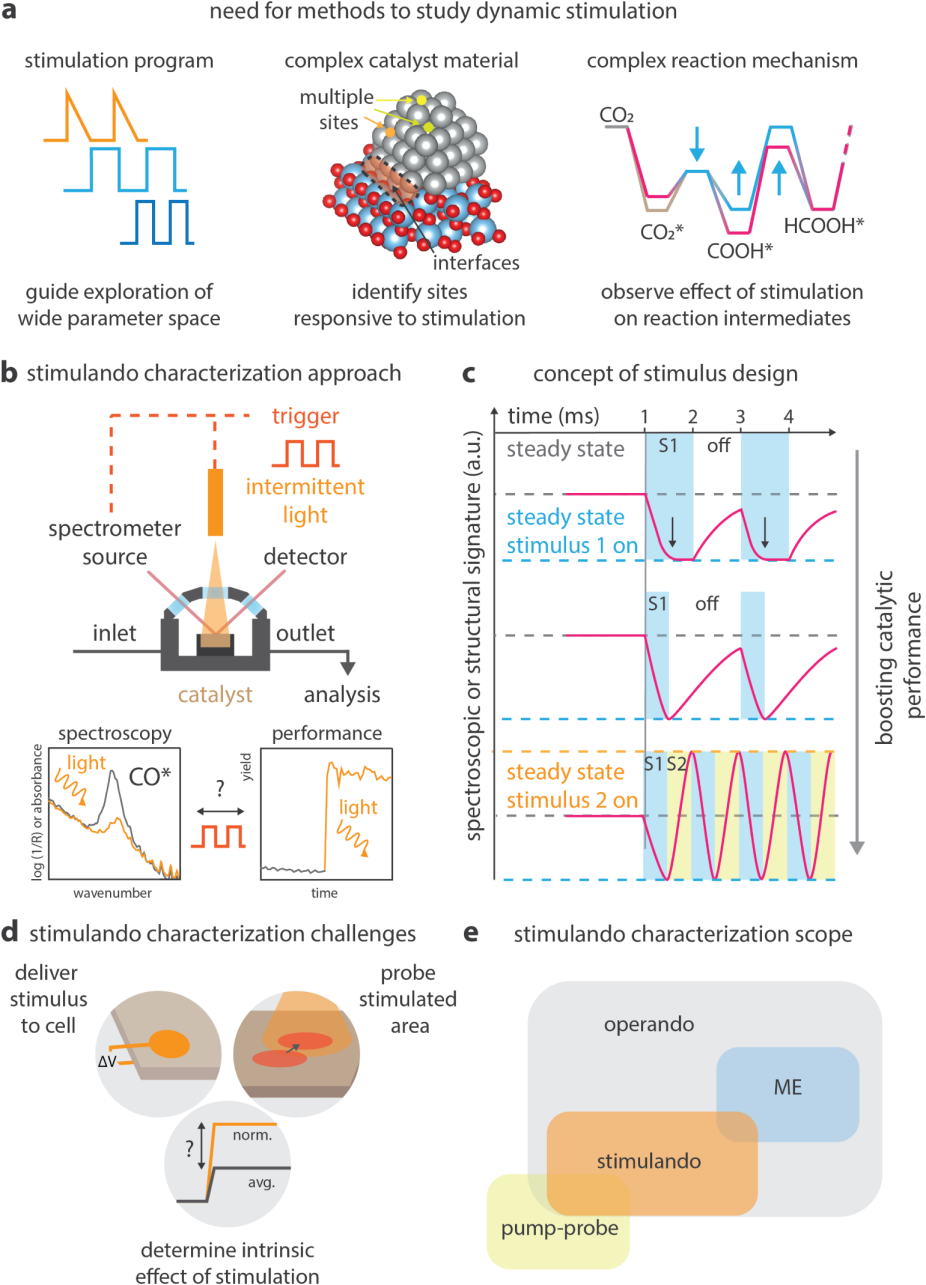
Understanding the mechanisms of dynamic catalyst stimulation. (a) Need for methods to understand dynamic catalyst stimulation. (b) *Stimulando* characterization approach: catalysts are characterized during stimulation, under reaction conditions, while simultaneously measuring the catalytic performance, with the aim to understand and guide better stimulation. (c) *Stimulus design* concept: one wants to correlate the catalytic performance with a spectroscopic or structural signature (e.g., intensity and/or position of IR signals). Example of potential application: When stimulating a catalyst with a certain intermittent stimulus (S1), a steady state may be reached already during a part of the duty cycle. Arrows (top) indicate when the stimulus should be turned off, since keeping the stimulus on is not contributing to enhancing the catalyst performance. To speed up the second half of the duty cycle, a second stimulus can be introduced (S2, bottom). (d) *Stimulando* characterization challenges: (i) delivering the stimulus to the catalyst under working conditions, (ii) probing the stimulated area, considering the spot size and penetration depth of the characterization/spectroscopic method, (iii) determining the intrinsic effectiveness of a stimulation, considering the part of the catalyst affected, and correlating this with spectroscopic trends. (e) A schematic overview of the scope of *stimulando*, operando, and representative dynamic operando approaches in catalyst characterization.

Such additional requirements introduce peculiar challenges that are not relevant to operando characterization and reflect a different philosophy and strategy for improving catalytic performance (Figure 10c,d). We propose naming such an approach *stimulando* characterization (echoing the term operando), which we define as “an analytical approach in which catalytic materials are characterized during stimulation, under reaction conditions, while simultaneously measuring the catalytic performance.” *Stimulando* is therefore a subfield of operando and shares some of its methodologies (Figure 10e). However, the two approaches differ in their primary aim: *stimulando* characterization is aimed at understanding stimulus-performance relationships and directing stimulus design, i.e., making the stimulation more effective for a certain catalytic device and reaction. On the other hand, operando characterization aims to establish structure-performance relationships of catalysts and yield information about mechanisms.

*Stimulando* and ME spectroscopy approaches share the use of periodic, intermittent changes in the reaction conditions and may at first appear analogous. However, they are also distinct in their goal and methods. In ME spectroscopy experiments, one wants to identify reactive and spectator species in a certain catalytic reaction. Usually, the reactor atmosphere is changed periodically by varying the gas composition to perturb the steady state of the catalyst and observe the dynamic change in surface coverage of different species. Change in modulation—for example, by switching from one gas to the other or from a mixture to just one gas—is done with the purpose of studying the behavior of surface species in different environments. The modulation is not changed to have a certain effect on catalysis. On the other hand, in *stimulando* spectroscopy experiments, one wants to understand how the catalyst is affected by the stimulation to then change the stimulation itself in different ways (frequency, amplitude, waveform, duty cycle, etc.) to boost catalytic performance.

*Stimulando* spectroscopy may also be used to interrogate reaction mechanisms proposed by theory and operando spectroscopy approaches. For example, it was predicted that stimulation of Ru-based catalysts by dynamic charging would result in different degrees of enhancement of methane steam reforming performance, depending on which of two proposed reaction mechanisms was assumed to take place.^33^ Provided that such a theoretical model and a stimulation experiment can be meaningfully compared, one could use catalyst stimulation as a means to test and possibly falsify the proposed reaction mechanisms. In other words, *stimulando* characterization techniques can add a new handle to unravel catalytic mechanisms and identify the nature of active species and sites.

The seemingly subtle differences between *stimulando* and operando approaches result in different challenges and call for the adaptation of the methods to understand catalyst stimulation. We believe the following aspects are important to consider when designing *stimulando* characterization experiments:

#### 7.3.1 Correlating Stimulation, Spectroscopy, and Catalytic Performance

The ultimate stimulated catalysis experiment would involve the stimulation of the entire catalyst. In practice, this will most probably not be the case, as the stimulation may affect only a part of the catalyst material or device. In this case, there are at least two important aspects to consider: First, the part, or region, of the catalyst that is stimulated and the part that is monitored by spectroscopy should (at least partially) overlap. For example, UV–visible light penetrates only a few (tens of) micrometers in most catalyst materials, while hard X-rays are transmitted through millimeter-thick samples (depending on energy and catalyst composition). Therefore, in an X-ray absorption spectroscopy experiment where UV–vis light is used to stimulate the catalyst, only a part of the sampled material will be affected by the stimulation, complicating interpretation of the results. This challenge is common to operando photocatalytic experiments, with the difference that here light is used to modulate catalysis rather than to drive the chemistry. To mitigate this issue, X-ray fluorescence might be used to collect a signal from the sample surface. Second, to yield meaningful insights into stimulus-performance relationships, the change in catalytic performance upon stimulation should be evaluated against the fraction of catalyst that is stimulated. Therefore, in principle, the extent of the stimulation itself should be measured in control experiments and/or modeled to properly design and interpret experiments.

#### 7.3.2 Low Surface Area of Catalytic Devices

Dynamically stimulating a catalyst material introduces constraints in catalyst design related to delivering the stimulation where it is needed. Often, a catalyst device is produced, where the catalyst is in the form of a thin film that can be stimulated during reaction. For example, in catalytic condenser devices, a thin film of catalyst is deposited on one of two conductive plates that are separated by a thin layer of dielectric material. In the case of dynamic strain, surface acoustic wave devices are often used, where a thin layer of catalyst material is deposited on a ferroelectric crystal. Such methods allow (at least in principle) stimulation of the whole catalyst, provided the catalyst layer is thin enough. However, they introduce the challenge of limited surface area—mostly geometrical, due to the absence of porosity—in the context of *stimulando* spectroscopy experiments. This in turn results in low signal-to-noise ratios for a given coverage of a certain intermediate or concentration of a species. To address this issue, one may use surface-sensitive techniques, such as attenuated total reflection infrared spectroscopy (ATR-IR), and/or tip-enhanced techniques, such as photoinduced force microscopy (PiFM) and tip-enhanced Raman spectroscopy (TERS). However, each technique has its subtleties and may be more or less compatible or interfere with a given stimulus. For example, dynamic strain would result in vibration of the sample, which would make tip-based spectroscopic methods extremely challenging, if not incompatible. Light stimulation may also interfere with Raman-based methods depending on the relative frequency of probing and stimulating light. Additionally, one can revert to lock-in amplification methods to improve the quality of the spectra.

#### 7.3.3 Cell Design

Dedicated cells are needed in order to dynamically stimulate catalysts while measuring catalytic performance under relevant conditions and acquiring spectra of good quality. For this, valuable lessons can be learned from operando spectroscopy, where cell design has been improved over decades.^268^ The main additional requirements are that (i) the catalyst must be stimulated while at work inside the cell without disrupting spectroscopic and catalytic measurements and (ii) the catalyst is stimulated in the region probed by spectroscopy. The cell design needed to bring the stimulus to the catalyst will depend on the specific method of stimulation used: for *stimulando* characterization during charge and Joule heating stimulation, electrical contacts and insulation must be added to the cell, while for light stimulation, one needs a (set of) window(s) transparent to both the stimulus and the spectroscopic probe. For example, a Harrick cell was used to study UV–vis stimulation during Diffuse Reflectance Fourier Transform Infrared Spectroscopy (DRIFTS), using a dome equipped with two IR-transparent windows and a quartz window for UV–vis light stimulation.^270^ Finally, since many catalyst devices for dynamic stimulation are in the form of thin films, the cell design should be aimed at minimizing external diffusion limitations and preferential gas flow. Inspiration can be taken from existing (micro)­reactor design.^271–273^

#### 7.3.4 Temporal Resolution

In *stimulando* characterization experiments, one would ideally want to follow changes in catalyst chemistry at the time scale of the stimulation, gain information on which species react to the stimulus, and how fast they do so, to ultimately change the stimulation accordingly. Nonetheless, information on average properties of the system in time (and space) is still very valuable. For example, the change in time-averaged coverage of CO during stimulated CO oxidation can be monitored using traditional vibrational spectroscopy techniques. A change in CO coverage from steady state to a certain limit cycle should result in different CO IR time-averaged spectra, leading to band broadening and a change in intensity (potentially influenced by vibrational coupling effects).^274^

Time-resolved measurements with chemical specificity, such as transient IR or Raman spectroscopy, will be key to better understanding stimulated reaction mechanisms and finally guiding catalyst and stimulus design. Many spectroscopic methods can be integrated with dynamic stimuli, including light, electricity, and heat. Figure 11 presents an overview of common spectroscopic and analysis techniques and their respective temporal resolution, compared with the window relevant to dynamic and stimulated catalysis. In time-resolved stimulated experiments, one may be able to identify short-lived reaction intermediates, which are otherwise going undetected due to their transient behavior. Such a prospect is exciting, as it would yield new insights that are relevant in the broader context of steady-state catalysis. When using pulsed light as a stimulus, ultrafast measurements on the femtosecond and picosecond time scales could be envisioned to better understand bond-making and -breaking processes, charge transfer, and energy transfer.

**Figure 11:**
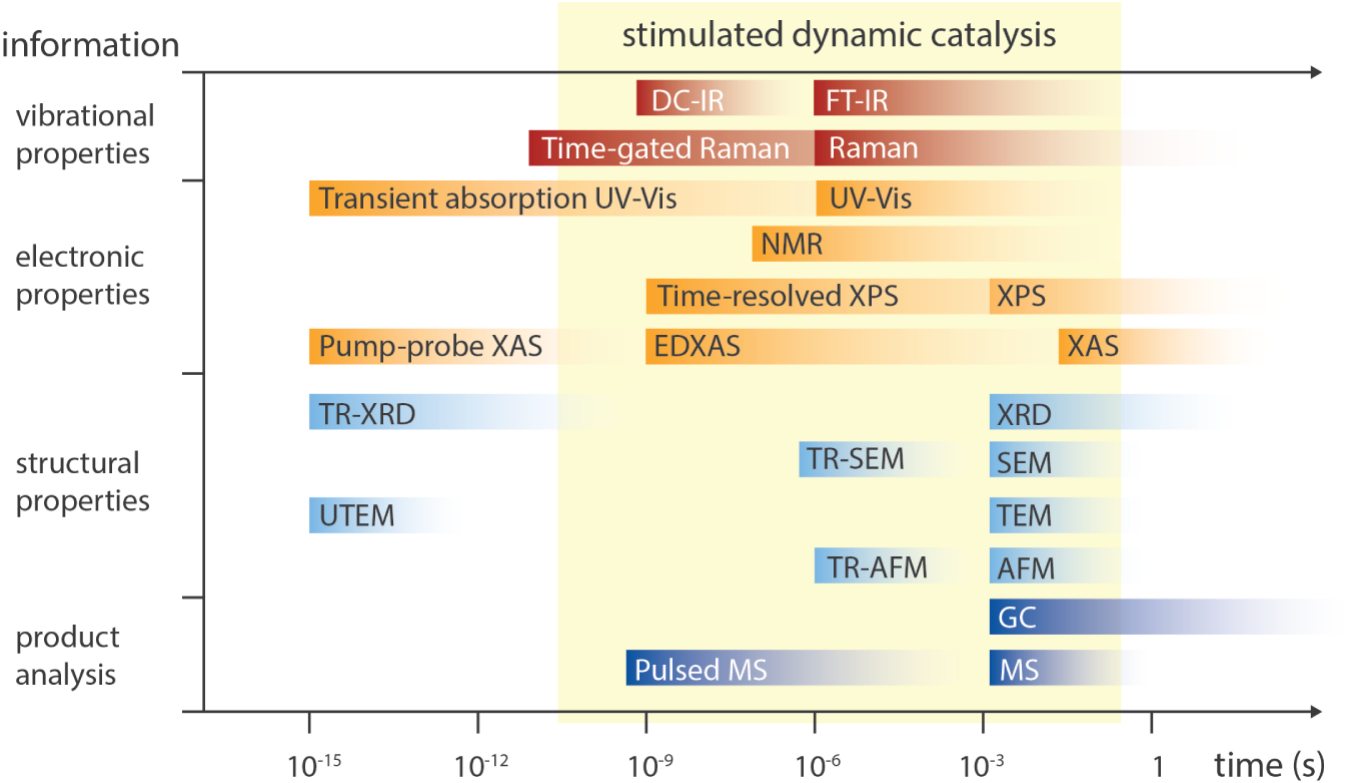
Overview of analysis techniques probing vibrational modes, electronic states, sample morphology, or catalytic performance ordered according to temporal resolution. For comparison, the typical time scales of stimuli used in resonant catalysis are indicated. DC-IR: dual-comb infrared spectroscopy; FT-IR: Fourier-Transform IR spectroscopy; UV–vis: ultraviolet–visible spectroscopy; NMR: nuclear magnetic resonance spectroscopy; XPS: X-ray photoelectron spectroscopy; XAS: X-ray absorption spectroscopy; EDXAS: energy-dispersive XAS; TR: time-resolved; XRD: X-ray diffraction; SEM: scanning electron microscopy; TEM: transmission electron microscopy; UTEM: ultrafast TEM; AFM: atomic force microscopy; GC: gas chromatography; MS: mass spectrometry.

#### 7.3.5 Catalyst Stability and Stimulation Reversibility

To reach a satisfactory signal-to-noise (S/N) ratio, many time-resolved spectroscopic techniques rely on averaging signals acquired at different phase delays with respect to a trigger or a pump across multiple periodic stimulations. For example, in step-scan IR spectroscopy, the movable mirror of the Michelson interferometer moves in discrete steps, and time-resolved IR intensity values are recorded at each step, corresponding to a specific point in the interferogram. The interferograms are then reconstructed using intensity data at each time interval for every mirror position after averaging over multiple stimulation cycles. The time-resolved spectra are finally obtained by Fourier transform, with up to 1 ns time resolution.^275^ It is important to note that the resulting spectra will be affected by any nonperiodic change in spectral features (e.g., band intensity, position, background), which might be induced by irreversible changes in the catalyst material structure or surface chemistry. For a proper interpretation of the spectra, it is therefore crucial that the catalyst remains stable over time for multiple stimulation cycles (on the order of hundreds or thousands depending on the required time resolution and S/N ratio). Any structural changes or deactivation during the measurement would make the results difficult or unreliable. To address this issue, the key recommendation in experimental design is to first verify that the catalyst remains stable over time and that its response to stimulus pulses is consistently similar. This is, in general, easier to achieve for bulk time-resolved techniques than for single-atom analysis such as time-resolved TEM, as nanoscale structural changes are rarely fully reproducible and repeatable.

#### 7.3.6 *Stimulando* Microscopy and Spatial Resolution

Dynamic stimulation of catalysts can, in principle, be performed both in time and in space, for example, by scanning a laser on a catalyst surface. It is known that even in nonstimulated catalysis, the catalyst structure at different scales can have a crucial role in surface chemistry and performance.^250,276,277^ During reaction and stimulation, structure–property relationships of components and their dynamic cooperation can influence processes at different length scales, which require microscopy monitoring beyond ensemble-averaging analyses. For example, photoemission electron microscopy (PEEM) was used to study the effect of forced carbon monoxide partial pressure oscillation on the suppression of chemical turbulence in CO oxidation over Pt(110) by delayed feedback.^278^ Addition of stimuli, such as light-induced in situ TEM or the use of the imaging probe itself as a stimulus, is not a routine application in microscopy and presents new challenges for established microscopy methodologies.^279^ Most microscopy techniques fall short in their acquisition frame rate below milliseconds and can only observe average effects of the stimulus on the catalytic system. Further development to integrate different stimuli, their high-frequency modulation, and correlated ultrafast imaging, e.g., UTEM at subpicosecond time resolution, is a challenging perspective. Depending on the complexity of a catalytic system, e.g., single crystal surfaces, supported nanoparticles, or pelleted powder, the stimulated volume must be characterized in all three dimensions to maximize and compare the efficiency of stimulated catalysis.

#### 7.3.7 Interference Stimuli and Probe

Ideally, in *stimulando* experiments, a photon/electron probe is used solely to investigate the ultrafast structural dynamics of the catalyst and reaction intermediates. However, high-energy probes, such as hard X-rays (e.g., X-ray Free-Electron Lasers) and electrons at high accelerating voltages (e.g., Ultrafast Transmission Electron Microscopes, UTEM), can introduce additional undesirable stimuli to the studied catalytic systems. Such undesired effects range from local sample heating to beam-induced damage, leading to irreversible structural changes in the catalysts (e.g., amorphization).^280^ Therefore, similar to in situ and operando practices, the optimal dose rate and/or total dose for a *stimulando* experiment can be determined by continuously probing the catalyst’s structure, first without stimuli and then with stimuli (applied both continuously and periodically). In the case of UTEM, minimizing the dose rate and total dose can be achieved by decreasing the electron current density (e.g., to an electron per pulse) and limiting the number of excitation/detection cycles. To address the resulting low signal-to-noise ratio, the use of direct electron detectors capable of detecting individual electrons is highly advantageous.

#### 7.3.8 Developing Ultrafast Heating as a Diagnostic Tool

Ultrafast heating methods can serve as diagnostic tools to probe reaction dynamics at time scales that are relevant for surface reaction kinetics but typically difficult to observe. Indeed, temperature jump experiments with an IR laser enabled heating of a zeolite catalyst in nanoseconds, where higher temperatures (up to a few hundred K) were sustained until several tens of microseconds on the catalyst surface. The system can cool down within several milliseconds, depending on the material’s thermal properties.^258^ Gaining control over the surface temperature in nanoseconds to microseconds allows both mechanistic and dynamic investigation of the reaction on the catalyst surface, which is not possible with the current time-resolved techniques with conventional heating in this time regime. Observation of surface events such as desorption and readsorption at even shorter time scales (tens of ps) was possible by heating the metal surface with femtosecond lasers.^281^ Pulsed Joule heating was also used to study kinetics, for example, in cellulose fragmentation.^282^ Advancing these tools can deepen our understanding of intrinsic catalytic mechanisms and limitations, providing fundamental insights critical to optimizing dynamic or traditional catalysis driven by heat. Furthermore, understanding how dynamic heat generation impacts the system is essential, as heat is an inevitable byproduct in other dynamic catalysis domains, such as strain- or light-induced reactions.

## 8 “Fruit Fly” Reaction and Catalyst

In evolutionary, biomedical research, and adjacent fields, Drosophila, the common fruit fly, plays a critical role as a well-understood model system that allows research across fields and groups. Similarly, dynamic and stimulated catalysis will benefit substantially from one or more well-studied model systems. So far, the community has studied a variety of reactions, but a common ground has not been reached. Finding a “fruit fly” catalyst system would allow us to compare results between laboratories and between different stimuli. Here, we propose a few criteria and suggest a few systems that match most of these criteria.

1. The ideal reaction/catalyst pair would be well-studied in thermochemical catalysis so that the standard mechanistic pathways are known and predictive and interpretive modeling procedures can be easily adapted.
2. The reaction should have multiple possible products with different mechanistic pathways so that the direct influence of dynamic stimuli on selectivity can be assessed. This is important since changes in observed reaction rate alone are difficult to distinguish from unaccounted effects. Meanwhile, more complex reactions with more than a few possible reaction products should be avoided at first.
3. The intermediates should be long-lived, and the rate-limiting steps of different mechanistic pathways should have different time scales. This allows targeting of different time scales by external stimuli, thereby investigating different time domains. Moreover, compatibility with *stimulando* characterization methods would allow the study of the effect of stimulation on different reaction intermediates.
4. The reaction and catalyst should be affected by as many possible different stimuli as possible so that cross-correlations can be made between different modes of stimulation. Additionally, there should be a possibility to excite different components of the system (such as support and catalyst) with either the same or with different stimuli. This orthogonality of different stimuli, where each stimulus addresses different components of the system, will allow testing potential synergistic effects of multiple different dynamic stimuli.
5. The catalyst should be stable, and it should be straightforward to monitor the catalyst’s stability over time. In dynamic catalysis theory and modeling, reversibility is assumed. However, systematically scanning pulse parameters experimentally requires many hours. Thus, for a meaningful comparison between theory and experiment, stability is required.
6. The reaction should be accessible for laboratory research: the catalyst should have simple, robust preparation, should not be too expensive, and should be compatible with simple lab equipment. The reaction should occur at or near ambient pressure, require relatively low temperature (<400 °C), should not use or produce extremely toxic species, and the products can be measured with common tools such as spectroscopy, GC, and MS.

Although there is not a single catalyst system that fulfills all criteria, we suggest five possible candidates that are well suited. The proposed reactions target small molecules for the time being because of ease of operation, but once technologies mature, specialty chemicals and pharmaceuticals may be valuable targets.

**CO**_**2**_ **hydrogenation over supported catalysts** is a well-studied system that allows modulation between multiple products (CO, methanol, and methane), depending on reaction conditions and catalyst structure. These products arise from distinct pathways involving formate, methoxy, or carbide-like intermediates with different lifetimes and kinetic barriers. For example, in Cu/TiO_2_ catalysts, the metal and support can be addressed independently by using different modes of stimulation. Most notably, UV light preferentially excites the oxide support, while visible light can couple efficiently with Cu nanoparticles through plasmonic resonances, depending on their size. Dynamic charge injection, localized heating, mechanical strain, and optical excitation can each affect the Cu and TiO_2_ components differently in terms of electronic structure and adsorption energies, enabling orthogonal control. The catalyst system is stable and regenerable and operates below 300 °C at moderate pressures with product detection via GC or IR spectroscopy.

**Selective hydrogenation of acetylene on Pd** offers a clear product selectivity between ethylene and ethane through competing mechanistic pathways. Long-lived intermediates, such as surface-bound vinyl species and Pd hydrides, evolve on different time scales and respond differently to stimuli. Electric fields and light modulation have all been shown to influence selectivity, and plasmonic Pd structures can enhance light absorption. Catalyst formulations are stable and regenerable, and the reaction typically operates between 50–150 °C, making it easily carried out in standard laboratory reactors using GC for product analysis.

**Alcohol oxidation on Cu, Ag, Au, or Pt**, such as methanol or ethanol oxidation, involves stepwise transformations with multiple possible products, such as formaldehyde, acetaldehyde, acetic acid, and CO_2_. These pathways involve kinetically distinct and long-lived intermediates, such as formate and CH_3_CO species, allowing the impact of dynamic stimuli on selectivity to be explored. The metal nanoparticles and supports can be excited independently with light, heat, or electric fields. These systems are straightforward to handle in the lab, operating below 400 °C and analyzable with GC and FTIR.

**Ammonia decomposition on Ru or Ni** features a well-known dissociation mechanism involving NH_*x*_ intermediates that evolve stepwise to form nitrogen and hydrogen. These intermediates exhibit different kinetics and have been inferred through spectroscopic studies and transient experiments, enabling time-domain targeting, even though the final product is singular. The system can be influenced by electric fields, thermal cycling, strain, and potentially light when using nanostructured metals. Catalyst stability is high under inert conditions, and the reaction can be run at moderate temperatures with a simple gas-phase analysis such as TCD.

**Selective oxidation of methane to methanol and other oxygenates** has been a long-standing challenge in catalysis due to the difficulty of activating methane while avoiding overoxidation to CO_2_.^283^ Partial oxidation pathways proceed through CH_3_ and CH_3_OH intermediates, which are relatively long-lived compared to subsequent combustion steps. These intermediates offer opportunities for time-selective intervention with external stimuli to enhance methanol selectivity. Catalysts such as Cu- or Fe-exchanged zeolites, supported noble metals, and plasmonic systems have shown potential in influencing product distributions under thermal, electrical, or optical stimulation, though consistent selectivity remains limited. Reported examples often operate below 300 °C and at ambient or mild pressures.

### 8.1 A Final Note on Metrics

To evaluate the effect and efficiency of dynamic stimulation, different metrics can be used, which are summarized below and discussed in detail in previous work:^32^ (i) The turnover efficiency, η, defined as

3 $$\eta =\frac{{\overset{―}{TOF}}_{stim}-{\overset{―}{TOF}}_{ss}}{\nu_{stim}}$$

where ${\overset{―}{TOF}}_{stim}$ is the average turnover frequency during stimulation, ${\overset{―}{TOF}}_{ss}$ is the turnover frequency without stimulation at the steady state (SS), and ν_stim_ is the stimulation frequency. This is an indication of the intrinsic efficiency of the stimulation, where, in the best-case scenario, every cycle of the intermittent stimulation causes a catalytic event at every surface site. (ii) The effective catalytic rate, defined as

4 $${\mathrm{r̅}}_{eff}=\eta {\overset{―}{TOF}}_{stim}$$

A higher effective catalytic rate indicates that input energy oscillations are more effectively used to convert reactants into products. (iii) The overall efficiency of the stimulation is another important metric to be considered, which will ultimately determine the viability of dynamically stimulated catalysis. For this, the energy balance of the system should be calculated and compared to nonstimulated catalysis as a benchmark.

## 9 Conclusion and Outlook

In conclusion, stimulated dynamic and resonant catalysis proposes a fundamental shift in the way catalyst performance can be designed and controlled, moving beyond steady-state kinetic boundaries and equilibrium thermodynamics. The goal is to improve catalytic performance in ways that static catalysis cannot achieve. The strategy of dynamic and resonant catalysis is to use programmable stimuli such as light, heat, charge, and strain to intermittently change surface coverage and active sites on time scales comparable to catalytic turnover. This poses challenges for theory and experiment alike to understand, predict, and realize stimulus-driven and time-dependent changes in catalyst function. While evidence of improved catalytic performance under dynamic operation is growing, new methods and frameworks are needed to tap the full potential of a stimulated catalyst operation.

Dynamic catalysis demands models that can capture the transient, stimulus-driven evolution of surface coverages and surface energies of reaction intermediates and transition states. Stimulus-dependent microkinetic models, molecular dynamics, and catalytic resonance theory are beginning to offer insights into the mechanisms of stimulated catalysis and into nonequilibrium phenomena such as energy ratcheting and resonance-induced rate enhancements. However, achieving predictive accuracy will require careful uncertainty quantification and the treatment of model assumptions. For instance, better fundamental understanding is needed about: (i) how to account for stimulus interaction with the catalyst at various length and time scales (physical model and its effect on the chemistry, magnitude of the effect, distribution over the catalyst); (ii) the validity of the instantaneous switching assumption; (iii) the use of BEP relationships to predict transition states, which may fail for stimuli that can break scaling relationships, such as uniaxial strain and electric fields; and (iv) the validity of the elementary ratchet model to predict complex multistep reactions.

The models of dynamic catalysis predict that catalytic turnover can be enhanced by orders of magnitude, and selectivity can be steered to different products when the catalyst is stimulated so that (i) the binding energy of reaction intermediates changes by at least 0.2 eV (but desirably in the order of 1 eV) to yield effective catalytic ratchets and/or (ii) the surface coverage of reaction intermediates changes considerably from steady state, for example, by fast heating and cooling (rates in the order of 10^2^–10^9^ K/s); (iii) the stimulation is carried out at a frequency higher than the steady-state turnover frequency, usually in the order of 10^2^–10^6^ Hz, with duty cycles that can vary widely depending on the intrinsic kinetics of each catalytic state.

While theoretical models predict significant gains through changes in surface coverage and intermediate binding energies, experimental results have yet to reach such dramatic improvements. We believe this is due to two challenges: (i) the demanding conditions for effective stimulation and (ii) the complexity gap between models and real-world catalysts.

The theoretically predicted stimulation parameters pose significant challenges to realizing the potential of dynamic catalysis, even in laboratory settings. Much effort has been put into developing stimulation methods and catalytic devices that can meet the requirements of magnitude and frequency needed for effective dynamic stimulation. For example, catalytic condensers were shown to modify binding energy by fractions of eV at kHz frequencies and at temperatures up to 300 °C, paving the way for charge-stimulated dynamic catalysis. However, more effort is needed to demonstrate the application of catalytic condensers in boosting catalytic reactions. On the other hand, several examples of dynamic light- and heat-stimulated catalysis can be found, in part due to the relative ease with which such stimuli can be applied to catalysts. Nonetheless, challenges remain because of limited light penetration in catalytic materials on the one hand and heat dissipation during cooling on the other. Finally, the effect of dynamic strain on catalysis is yet to be understood and leveraged, despite decades of research in surface acoustic wave devices and the mechanical deformation of catalyst bodies.

Many catalytic reactions proceed via reaction networks involving multiple elementary steps, each with distinct time scales and a potentially different response to stimulation. Gaining a better understanding of the dynamic stimulation of catalysts will require advanced analytical and characterization methods. We propose here the concept of *stimulus design* as a complementary strategy to traditional catalyst design that aims to optimize the effect of stimulation on catalysis. Achieving this requires advanced *stimulando* characterization, a novel operando-inspired approach that characterizes catalysts under simultaneous reaction and stimulation conditions while monitoring the performance. Such an approach must overcome challenges related to spatial overlap of stimulus and probe, limited surface area of catalyst devices, temporal resolution, and ensuring catalyst stability under repeated cycling. Additionally, adapting in situ and time-resolved methods—such as IR, Raman, or ultrafast spectroscopies—and cell designs will be critical to revealing stimulus-induced changes at relevant time and length scales. Developing such a toolbox will be challenging, but it holds promise to guide effective catalyst stimulation to finally achieve transformative catalytic performance.

In principle, different stimuli (e.g., light, strain, or charge) can result in different changes in surface chemistry (e.g., different γ, δ values, different species being stimulated) and therefore different catalytic performance under dynamic stimulation. We anticipate that combining different stimuli can result in further improved catalytic performance, which may be fundamentally not achievable with one stimulation alone. We imagine complex multistep reactions where each optical pulse is designed to carry out one step and address only a specific subpopulation of reactants or intermediates. For example, one can imagine combining dynamic light—to excite one species preferentially (e.g., CO adsorbed on metals)—and dynamic charge—to modulate the binding energy of polar adsorbates the most. Playing with the relative intensity, frequency, duty cycle, and phase delay between the dynamic stimuli, one could impart tailored programs to modulate the surface chemistry in complex ways. It is possible that this approach moves us out of the realm of statistical chemistry and into a more deterministic regime, and certainly, it is chemistry far from equilibrium. Multistimulation introduces exciting new handles to steer catalytic performance, but it also introduces a virtually infinite number of stimuli combinations. We foresee that machine learning and automation will play critical roles in the exploration of such vast parameter space.

While in its infancy, the field of stimulated dynamic catalysis is rapidly growing and showing promise for the development of new catalytic technologies. The acceptance of such technologies will depend on multiple factors, such as benefit/cost considerations in both the implementation and operation phases. Down the line, we foresee the following possible hurdles to industrial implementation: (i) cost of the catalyst device per ton of product over the catalyst lifetime, compared to state-of-the-art; (ii) achievable scale, in terms of space-time yield of a product; (iii) need for robust materials, resistant to mechanical fatigue, thermal degradation, or restructuring under sustained high-frequency dynamic operation; (iv) energy balance considerations, taking into account the energy needed for the stimulation and the energy saved by operating at milder conditions and/or simplifying downstream separations when selectivity is improved. Pushing stimulated dynamic catalysis over TRL 4 will require concerted efforts from academia and industry and entrepreneurial efforts, such as academic spin-offs.
